# Remote Deep-Ultraviolet Laser Ablation in Connection with Electrospray Ionization–Atmospheric Pressure Chemical Ionization (rDUVLAESCI): A Novel Dual Ionization Source for Molecular Mass Spectrometry

**DOI:** 10.1021/acs.analchem.4c04392

**Published:** 2025-01-22

**Authors:** Barbora Papoušková, Petr Fryčák, Filip Gregar, Karel Lemr, Tomáš Pluháček

**Affiliations:** Department of Analytical Chemistry, Faculty of Science, Palacký University Olomouc, 17. Listopadu 12, 77146 Olomouc, Czech Republic

## Abstract

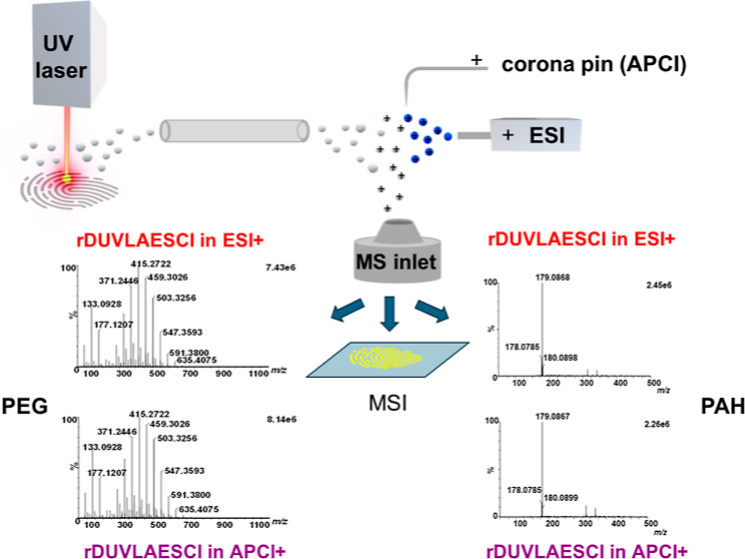

A novel remote deep ultraviolet laser ablation inlet connected to a dual electrospray ionization–atmospheric pressure chemical ionization (rDUVLAESCI) source is presented. This system allows for the simultaneous and spatial acquisition of mass spectrometry (MS) data for organic molecules with diverse polarities and molecular weights. Deep 193 nm UV laser ablation was used to sample analytes from dried spots for molecular MS analysis precisely. Furthermore, molecular MS imaging (MSI) with a variable laser spot size down to 3 μm was demonstrated. The complementary ionization modes generated mass spectra with sufficient analyte signals, detecting a broad range of molecules from polar compounds like caffeine and PEG 600, to nonpolar analytes, such as anthracene and wax esters, all within a single analytical run. Detection limits were found in the tens of attomoles per ablated/desorbed pixel. The powerful capabilities of the fully automated rDUVLAESCI dual source were demonstrated by visualizing the spatial distribution of new psychoactive substances on latent fingerprints. MSI for both sebum components and psychoactive substances revealed a connection between the chemical evidence and biometrical information. The rDUVLAESCI–MSI enabled the unambiguous identification of individuals, even using partially overlapped latent fingerprints. This unique rDUVLAESCI approach, with its remote laser ablation unit, improved spatial resolution and analyte coverage, particularly for nonpolar compounds.

## Introduction

Ambient mass spectrometry (MS) has become a powerful tool for analyzing a wide variety of molecules under native conditions.^1^ One of the most advanced ambient techniques is represented by laser ablation/desorption followed by the post-ionization of desorbed neutral particles by anionization source, namely, electrospray ionization (ESI), atmospheric pressure chemical ionization (APCI), and atmospheric pressure photoionization (APPI).^2^ During the last two decades, considerable efforts have been devoted to the development of advanced laser ablation electrospray ionization (LAESI) source designs. Lasers with wavelengths ranging from the deep UV (DUV) to the far IR have been successfully coupled with ESI, APCI, APPI, and low-temperature plasma ionization (LTP) to ionize various molecules with significantly different polarity and molecular weight.^2−4^

In addition, UV and DUV laser ablation have attracted attention for their ability to focus the laser beam to small spots (low μm scale) and for the efficient absorption of short wavelengths by a wide range of molecules or sample surfaces, respectively. The efficient desorption/ablation of minute samples within a single laser pulse without any extensive analyte fragmentation^5−7^ has allowed the DUV-LAESI/APPI/LTP MS analysis/visualization of intact small molecules, metabolites,^8−12^ lipids,^5,10,12^ dyes/inks,^9^ or even proteins^13−17^ in various matrices ranging from model wet or dried spots^8,11,13,14,16^ and banknotes^9^ to biological tissue sections.^5,7,10,12,14,15,17^ To the best of our knowledge, there has been no publication describing universal ion source design simultaneously acquiring DUVLAESI and DUVLAAPCI signals within a single analytical run with a variable laser spot size at the μm scale for direct analysis as well as for imaging purposes.

This study is aimed at the development and characterization of a novel dual rDUVLAESCI ion source that facilitates simultaneous electrospray and chemical ionization. Its applicability covers comprehensive direct analysis as well as MS imaging of a plethora of organic molecules. The source features a new custom-built interface and enables the automatic acquisition of rDUVLAESCI (ESI and APCI modes) data in a single analytical run. The simultaneous dual-mode acquisition facilitates new possibilities for the straightforward analysis of complex samples containing analytes of widely varying polarity. Analytes ranging from polar organic molecules to aromatic hydrocarbons were effectively ionized. Moreover, the proposed design with a remote laser ablation chamber provides an excellent lateral resolution of 3–160 μm and analytical performance allowing the analysis of minute samples. MSI of the traces of new psychoactive substances (naphyrone, butylone, cathinone, and flephedrone) visualized latent fingerprints linking illicit drug abuse with the identification of individuals.

## Material and Methods

### Chemicals

Theophylline (≥99%), benzoic acid (≥99.5%), stearyl stearate (≥98%), anthracene, and caffeine (pharmaceutical secondary standard) were provided by Sigma-Aldrich, Germany. Butylone, cathinone, flephedrone, methedrone, and naphyrone were obtained from Lipomed, Switzerland. Ethyl palmitate (97%), ethyl stearate (>99%), polyethylene glycol 600 (PEG 600), polyethylene glycol 10,000 (PEG 10,000), squalene (>97%), and acetone (≥99.8, HPLC grade) were from Fluka, Germany. Isopropanol (≥99.9, LC–MS grade), acetonitrile (≥99.9, HPLC gradient grade), methanol (≥99.9, LC–MS gradient grade), and formic acid (≥99%, ACS reagent) were from VWR, Germany. Stearic acid and palmitic acid were from Lachema, Czechia, and heptane was from Penta, Czechia. All chemicals were of at least analytical-grade purity, if not stated otherwise. Milli-Q water with a resistivity of 18.2 MΩ cm was produced by a Milli-Q reference water purification system (Millipore, France).

### rDUVLAESCI Ion Source Setup

**Figure 1:**
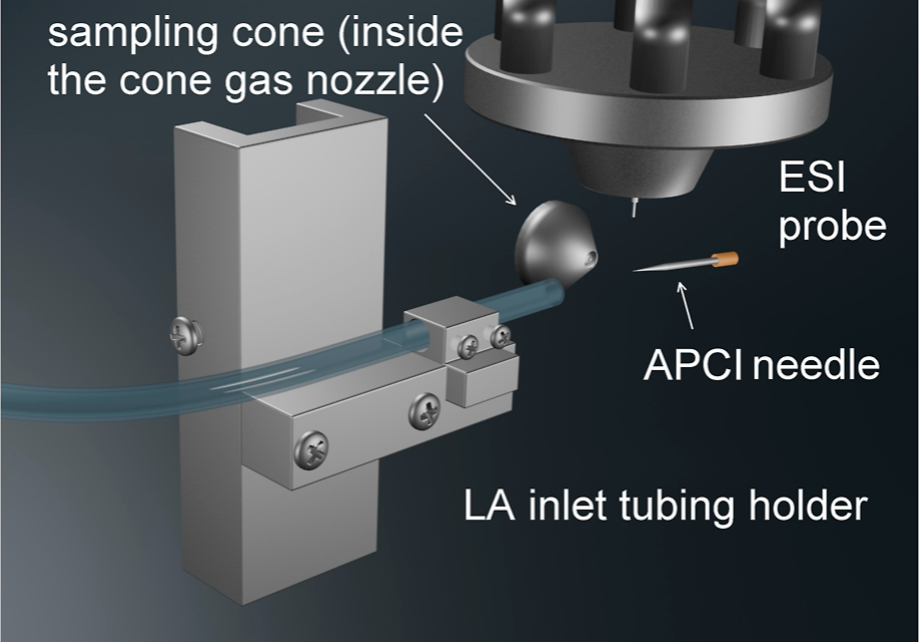
The rDUVLAESCI interface is mounted in the Waters ESCI Multimode ionization source. The cloud of ablated material is transported orthogonally toward the MS inlet into the ESI/APCI source.

The full description of the developed rDUVLAESCI simultaneous dual acquisition source is presented in the Supporting Information. Briefly, an Analyte G2 remote UV laser ablation unit (Photon Machines, USA) with a 193 nm excimer nanosecond laser was connected to a Synapt G2-S hybrid Q-TOF mass spectrometer (Waters Corporation, UK) via a proprietary interface designed for the commercially available ESCI Multimode ionization source (Figure 1). The optimized parameters for rDUVLAESCI–MS and rDUVLAESCI–MSI automated data acquisition within a single analytical run are summarized in the Supporting Information. The mass spectra collected from the spot analyses were evaluated using MassLynx 4.1 software (Waters Corporation, UK). Extracted time-resolved data from imaging experiments were visualized using the ILAPS software originally developed for laser ablation data reduction and imaging applications.^18^

### Preparation of Standards for Spot Analysis

A set of reference analytes differing in polarity and molecular weight was used to characterize the ionization properties. For each analyte, the stock standard solution of 1 mg mL^–1^ was prepared in either heptane (anthracene), isopropanol (butylone, cathinone, flephedrone, naphyrone, ethyl stearate, squalene, stearic acid, and PEG 600), acetone (stearyl stearate), or methanol (palmitic acid), based on solubility. A 2 μL aliquot was pipetted onto the microscopic slide and left to dry at laboratory temperature to create a spot with an average diameter of 14 mm.

### Preparation of Latent Fingerprints with Traces of New Psychoactive Substances

The two model latent fingerprints with trace amounts of new psychoactive substances were prepared to demonstrate the applicability of rDUVLAESCI–MS in molecular MSI. The following 5-step procedure was used to prepare minute volume samples: application of 50 μL of butylone and naphyrone (1 mg mL^–1^) on the microscopic slide (2.5 × 5 cm); spreading over the desired area; the complete drying of the slide at laboratory temperature; touching the microscope glass surface with a thumb; and a collection of latent fingerprints by printing on a new clean microscope glass. The second latent fingerprint sample with flephedrone and cathinone was prepared by using the same procedure with two donors placing their thumbs on the same microscopic slide to create partially overlapping fingerprints. The prepared latent fingerprints were subjected to MSI of endogenous and exogenous analytes using rDUVLAESCI–MSI. Personal identification was based on comparing characteristic dactyloscopic features of the drug and sebum-based 2D maps with the reference fingerprint visualized by a metallic dactyloscopic powder (bronze).

## Results and Discussion

### Characterization of the rDUVLAESCI Dual Ion Source

**Figure 2:**
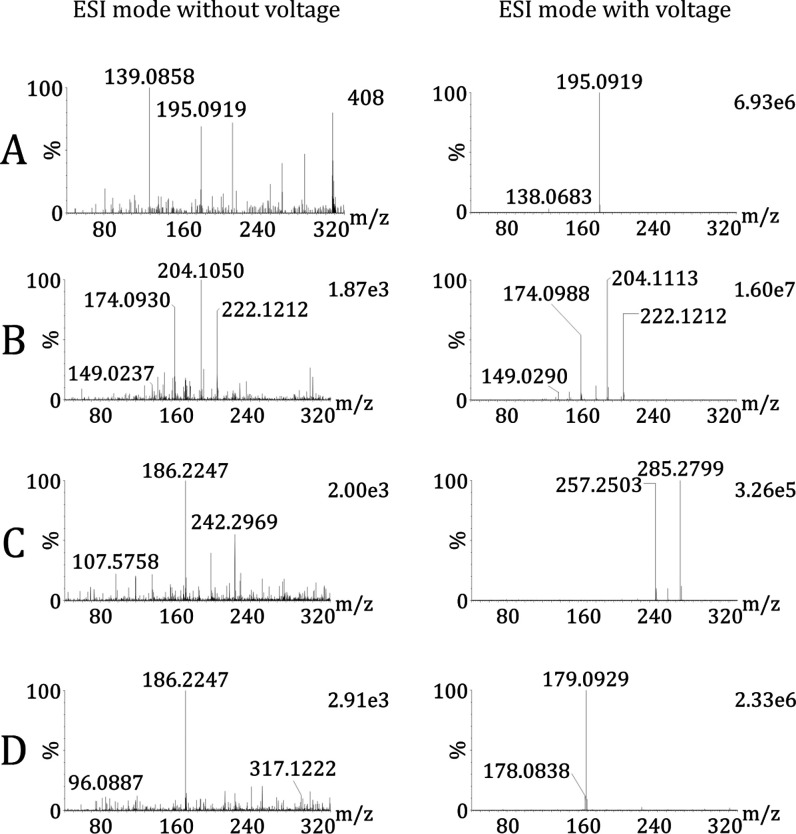
rDUVLAESCI–MS single line scan of dried spots testing the ionization efficiency of the laser itself (left panel) and the combination of laser ablation and ESI/APCI (right panel). rDUVLAESCI–MS analysis of dried spot (A) caffeine (*m*/*z* 195.0919); (B) butylone (*m*/*z* 204.1113, 222.1212); (C) ethyl palmitate (*m*/*z* 257.2503, 285.2799); and (D) anthracene (*m*/*z* 178.0838, 179.0929).

rDUVLAESCI ionization involves three consecutive steps: sample ablation/desorption, aerosol transport by helium, and ionization in the ESI/APCI dual source, where the generated charged particles/ions travel toward the MS inlet. The initial research focused on studying the DUV ionization of analytes spanning a wide polarity range, such as caffeine, butylone, anthracene, and ethyl palmitate (Figure 2). The contribution of DUV laser ionization to the total ion yield was found to be negligible (Figure 2, left panel). The rDUVLAESCI–MS signal intensities of anthracene and ethyl palmitate were up to 5–6 orders of magnitude and were not observed using solely ablation/desorption without applying voltage in the ESI/APCI source. For caffeine and butylone, the laser ablation/desorption alone generated a signal less than 0.02% of the maximum value obtained by rDUVLAESCI–MS. In addition, anthracene, a UV-absorbing aromatic compound, did not form a cation radical via photoionization (Figure 2). This confirms that ionization is primarily accomplished in the ESI/APCI source. Furthermore, the ESI and APCI mass spectra displayed low levels of fragmentation for the tested analytes as well as for fingerprint constituents (Tables 1 and S2). These findings demonstrate that optimized nanosecond DUV irradiation effectively facilitates complete desorption/ablation of the sample before ESI and APCI ionization with negligible contributions from photoionization (Figure 2).

**Table 1:**
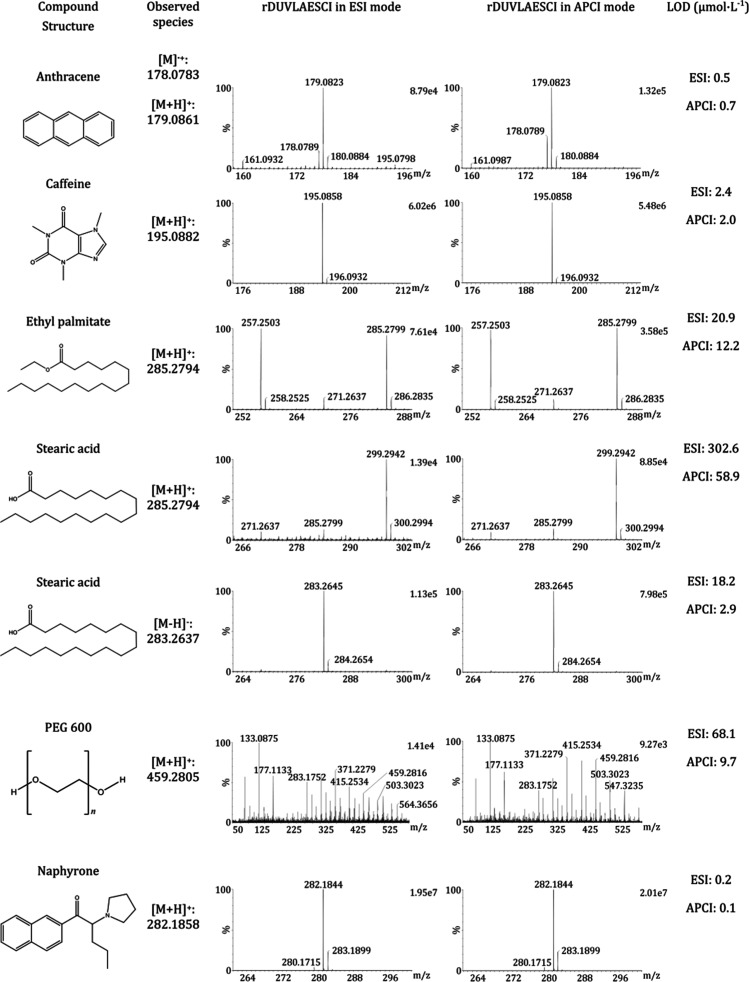
Overview of Molecules Used for the Novel Dual Source Characterization Together with LOD Estimation for rDUVLAESCI–MS in Both Modes, ESI and APCI

In general, no extensive fragmentation, greater than approximately 13%, was observed for the tested analytes subjected to rDUVLAESCI–MS analysis at the laser fluence of 1.21 J cm^–2^ (Table S1). The nondestructive character of the DUV ablation/desorption has already been verified for the tissue section analyses by Schäfer et al.^5^ Laser desorption ionization of bulk tissue samples using different laser wavelengths provided uniform spectra at 266 nm, 337 nm, 355 nm, 2.94 μm, and 10.6 μm, while no spectra were obtained at 532, 785, or 1064 nm. The ionization mechanism is primarily thermal, with laser energy causing tissue heating and water boiling, resulting in the formation of aqueous droplets and ions. Thermal ionization is preferred over photochemical ionization.^5^

**Figure 3:**
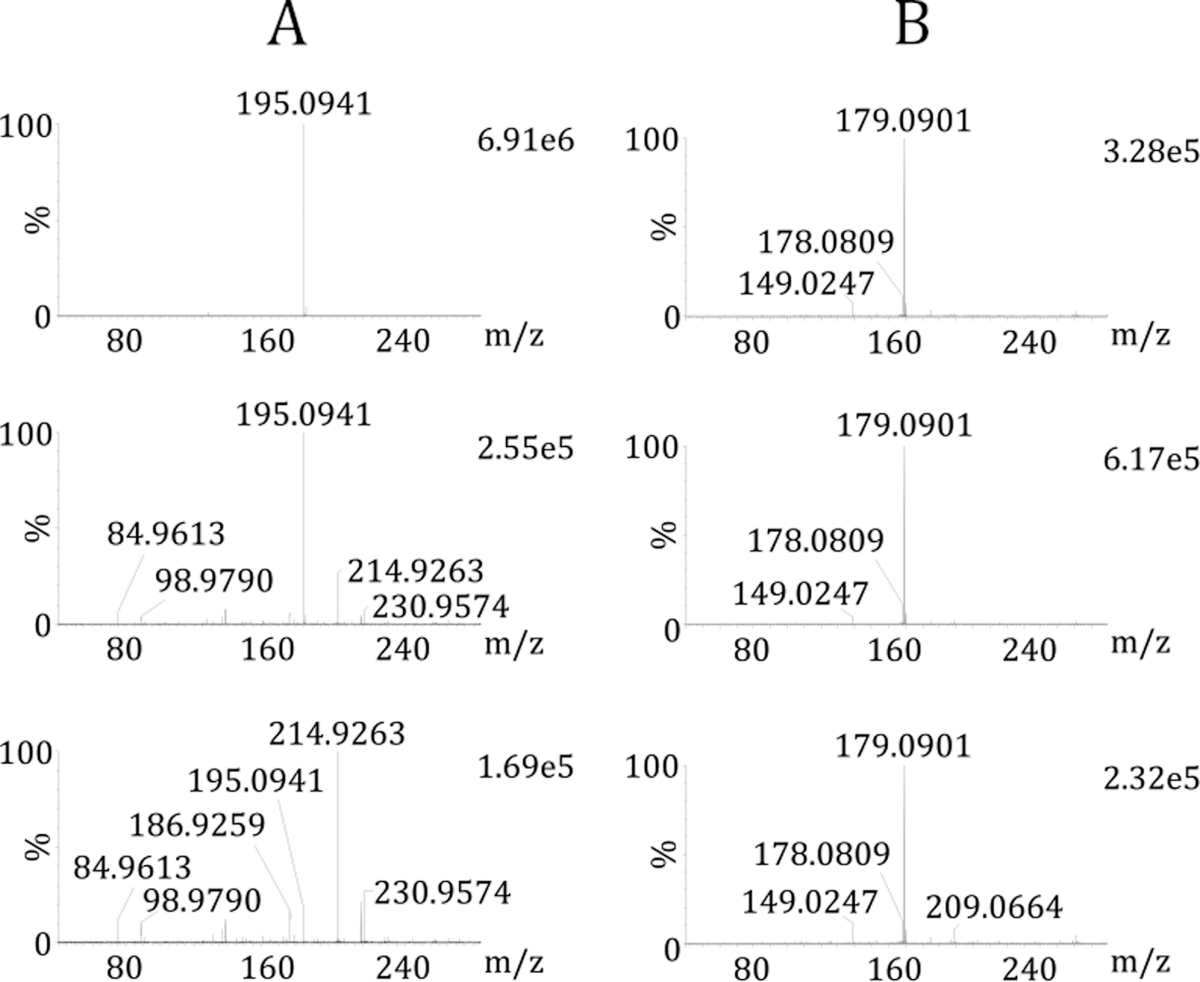
rDUVLAESCI–MS analysis with variable laser fluence. (A) Mass spectra of caffeine and (B) mass spectra of anthracene. The laser fluence of 1.21 J cm^–2^ (up), 6.05 J cm^–2^ (middle), and 12.1 J cm^–2^ (down).

Our results confirmed that the DUV nanosecond laser pulse did not cause the fragmentation of anthracene, caffeine, theophylline, benzoic acid, and palmitic acid even at high laser fluence up to 12.1 J cm^–2^, which is the fluence used for the efficient ablation/desorption of solid materials (Figure 3 and Table S1). Interestingly, even at the highest laser fluence, the ratio between the cation radical and the protonated molecule in the mass spectra of anthracene remained unchanged. The optimization of the ESI/APCI source parameters was focused on the capillary/corona voltage (3.3–4.3 kV for ESI and 3.0–4.0 kV for the APCI source), spraying liquid flow rate, and its composition to improve the signal-to-noise ratio (SNR). A significant impact of the spraying liquid flow rate on the ionization process was observed for both ESI and APCI modes. At higher flow rates (200 μL min^–1^), ESI generated more adducts, such as ammoniated molecules, and was prone to signal suppression or even signal loss. APCI produced cleaner spectra with fewer adducts. The difference was evident when analyzing ammonium-spiked olive oil as an example. The APCI revealed characteristic profiles of triacylglycerides (TAGs), diacylglycerides (DAGs), and free fatty acids (FFAs), while ESI generated only unrelated ammonium adducts (Figure S2). Therefore, further experiments were performed at a flow rate of 5 μL min^–1^ to obtain complementary mass spectra for spot and MSI experiments. Four spraying liquid compositions of water with 0.1% formic acid (FA), MeOH with 0.1% FA, MeOH/water 1:1 (v/v), and MeOH/water 1:1 (v/v) with 0.1% FA were compared using SNR for caffeine, butylone, ethyl palmitate, and anthracene. Although the highest overall signal intensity for rDUVLAESCI was achieved using a 1:1 spraying liquid of MeOH/water (v/v), the signal intensity for all tested compounds was quite similar, varying by less than an order of magnitude when using other spraying liquids. The most significant difference in signal intensities for rDUVLAESCI experiments was observed in favor of rDUVLAESCI in ESI mode for ethyl palmitate when using a spraying liquid MeOH/water 1:1 (v/v) with 0.1% FA (Figure S3). Interestingly, nonpolar/aromatic compounds are also easily ionized without any special demand on the spraying liquid composition. A signal for anthracene was detected using acidified water as a spraying liquid (Figure S3). Yet, the most intense and reproducible signals for all studied compounds were provided by the mixture of MeOH/water 1:1 (v/v), which was therefore selected for further experiments. The optimal ESI capillary and APCI corona voltage were set to 3.8 and 3.5 kV, respectively. The dried spots of anthracene, butylone, caffeine, cathinone, ethyl palmitate, ethyl stearate, flephedrone, naphyrone, PEG 600, squalene, and stearic acid were analyzed using rDUVLAESCI–MS to characterize the analytical performance for analytes significantly differing in the polarity (Tables 1 and S2). The dual source provided a unique ambient technique for detecting a wide range of analytes that are poorly ionized in standard ESI and APCI sources/modes. Both modes have comparable detectability at μmol mL^–1^ levels for anthracene, ethyl palmitate, caffeine, flephedrone, bytylone, and naphyrone. However, rDUVLAESCI–MS in APCI mode exhibited 2- and 7-times lower LODs for squalene, stearic acid (positive and negative ionization mode), and PEG 600 in positive mode, while rDUVLAESCI–MS in ESI mode showed approximately 2-times better detectability for some new psychoactive substances, namely, flephedrone and cathinone (Table S2). The lowest LODs were obtained for naphyrone, which corresponded to the detection of dozens of attomoles per pixel, making the rDUVLAESCI–MS useful for the analysis of minute samples.

### Tunable Lateral Resolution of the rDUVLAESCI Source for MSI

**Figure 4:**
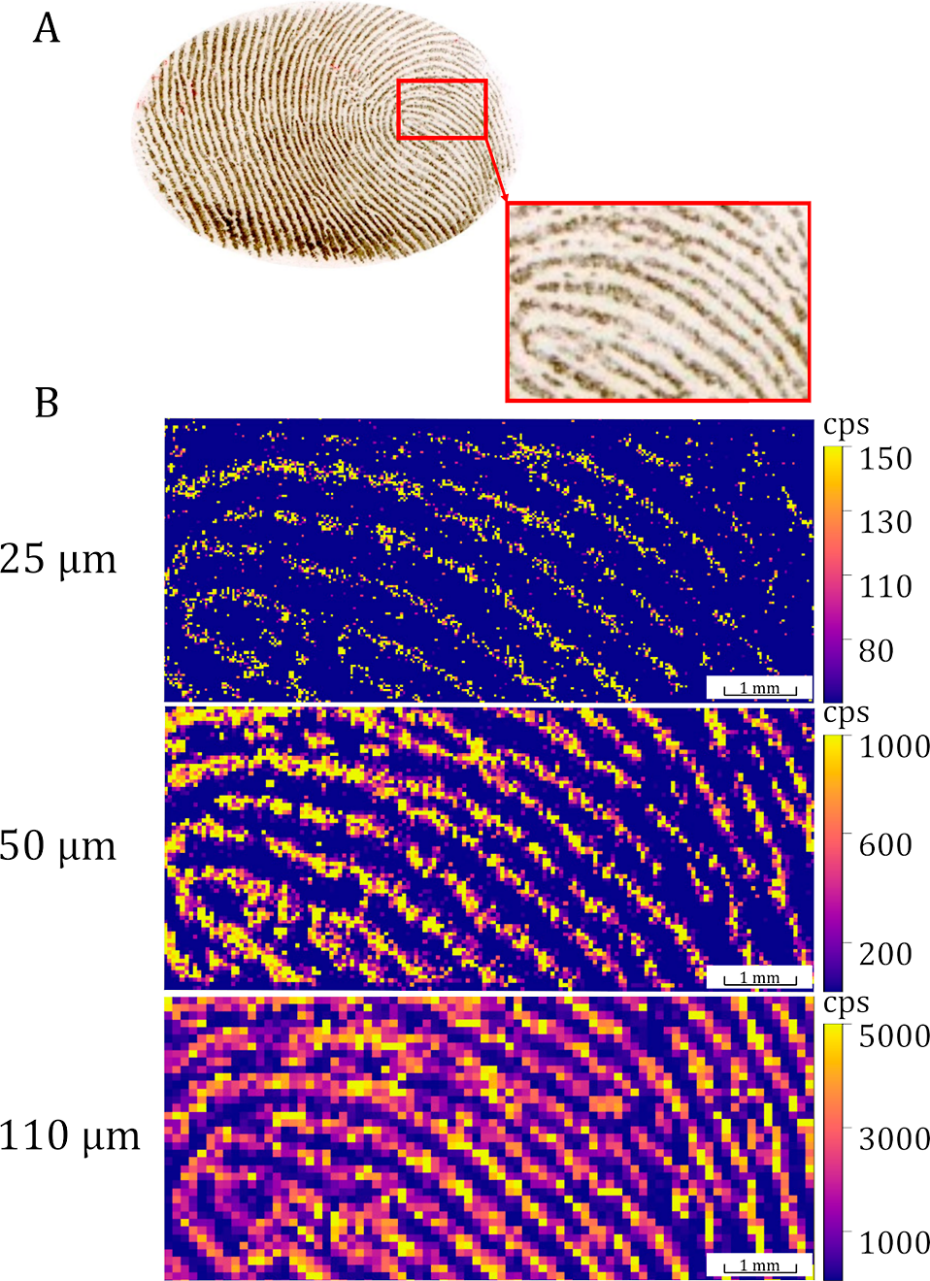
rDUVLAESCI–MSI using variable laser spot size. (A) Reference fingerprint visualized by bronze dactyloscopic powder and (B) rDUVLAESCI–MSI 2D images of squalene acquired at different pixel sizes: 25 μm (top), 50 μm (middle), and 110 μm (bottom).

The proposed rDUVLAESCI approach allows mass spectral acquisition with a variable laser spot size starting from 3 μm (Figure S6). The latent fingerprints containing psychoactive substances were subjected to the rDUVLAESCI–MSI. The remote laser ablation unit fitted with UV tunable laser power provides detailed insight into the molecular surface composition with a unique lateral resolution (Figure 4). The MSI with the highest lateral resolution revealed the spatial distribution of individual squalene features on each papillary line with an average size of 435.5 ± 57.4 μm.^19^ In general, the UV laser ablation does not impose any special requirements on the sample composition and surface; thus, the thin layer or thin cross-section samples (e.g., thin films and soft or even hard tissues) can be directly subjected to the rDUVLAESCI analysis. On the other hand, MSI using a remote ablation chamber depends on several factors (namely, beam size, repetition rate, scanning speed, and acquisition time) that should be adequately optimized to provide a reliable image quality without any distortion (such as smearing, blurring, etc.).^20^ The system’s reliability was tested using experiments with a slow laser repetition rate (1–7 Hz) and a fast acquisition time on the mass spectrometer (25 ms). This setup enabled the collection of 5–6 data points per sampling event, with sharp edges reflecting the laser pulses. Adequate wash-out time was confirmed, minimizing carryover effects (Figure 4), where no blurring was observed (well-separated signal for the papillary line and the interpapillary distance). The combination of high laser frequency, acquisition speed, synchronization of the LA pulse with MS acquisition, and short wash-out times (negligible carryover even for concentration-sensitive ESI sources) offers a promising approach for high-speed MSI with no apparent impact on the image quality or sample throughput. The applicability of MSI was demonstrated through visualization of the latent fingerprint, showing a potential link between unique chemical features and biometrical information.

### Analysis of Latent Fingerprints

**Figure 5:**
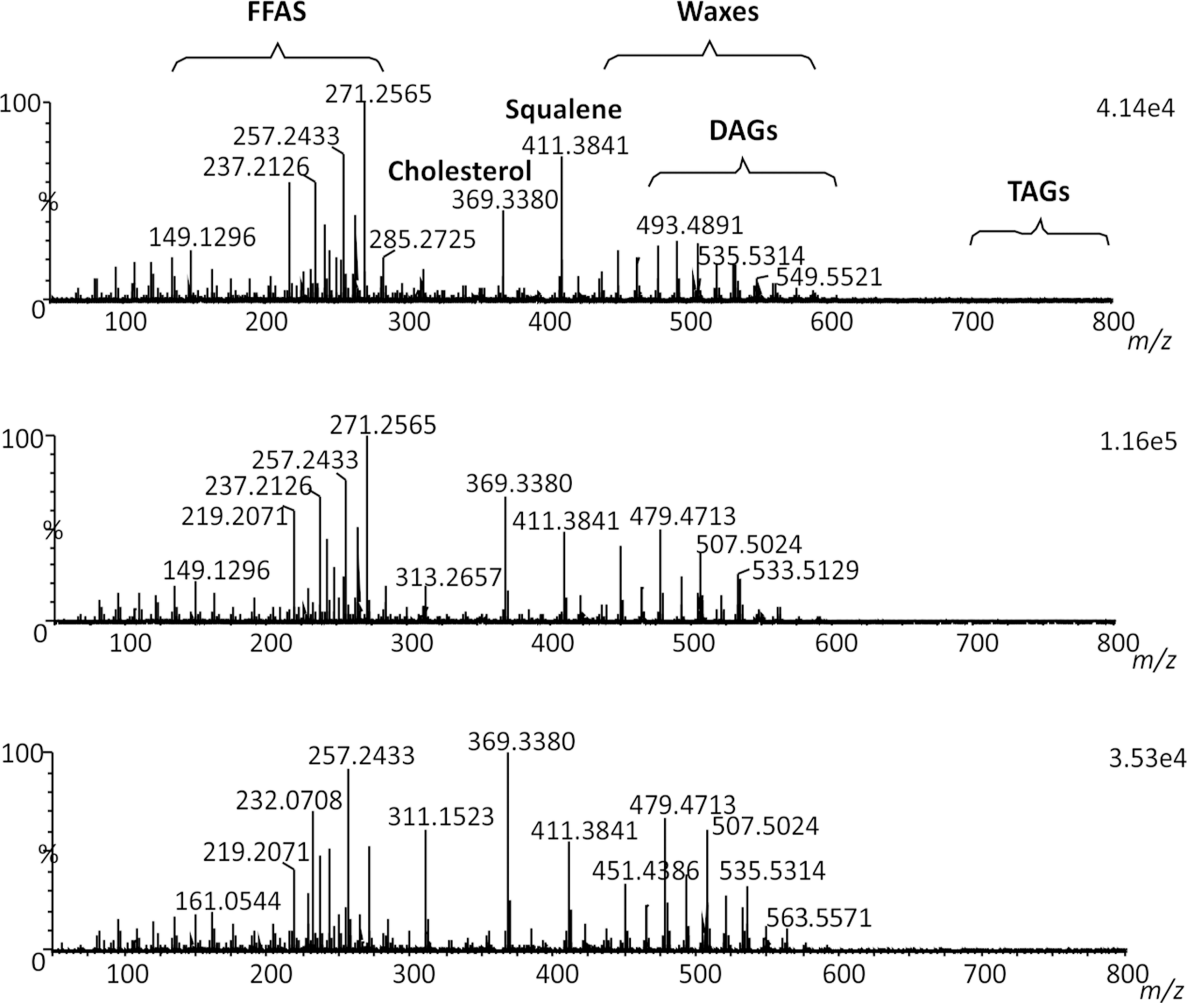
Characteristic fingerprint sebum-related compound detection (rDUVLAESCI mass spectra in profile mode without adjustment for accurate mass measurement). The small latent fingerprint sample amount generates the signal for all major constituents. Donor 1 (up); donor 2 (middle); and donor 3 (bottom).

Latent fingerprints are thin layers, averaging a thickness of 0.1 μm, composed primarily of sebaceous liquid, which contains mostly water and traces of FFAs, cholesterol, squalene, wax esters (WEs), DAGs, TAGs, and inorganic ions (such as Na^+^, K^+^, NH_4_^+^, etc.).^21,22^ The rDUVLAESCI–MS analysis performed on fingerprints collected from three donors revealed the presence of these characteristic organic compounds (Figure 5). The observed FFAs ranged from 12:0 to 31:0 and 16:1 to 18:1 and quite remarkably, protonated forms of FFAs were detectable under positive ionization conditions (e.g., sapienic acid). Further, cholesterol (rDUVLAESCI–MS in ESI mode: [M + H – H_2_O]^+^, *m*/*z* 369.3527, 1.6 ppm; rDUVLAESCI–MS in APCI mode: [M + H – H_2_O]^+^, *m*/*z* 369.3527, 1.6 ppm) and squalene (rDUVLAESCI–MS in ESI mode: [M + H]^+^, *m*/*z* 411.3997, 1.5 ppm; rDUVLAESCI–MS in APCI mode: [M + H]^+^, *m*/*z* 411.3988, −0.7 ppm), WEs with 28–42 carbons in length, including both saturated and unsaturated species (DB:1 and DB:2), both saturated and unsaturated DAGs ranging from 26:0 to 42:0, 25:1 to 36:1, 26:2 to 36:2, and 28:3 to 29:3 and TAGs ranging from 36:0 to 55:0, 36:1 to 54:1, 38:2 to 54:2, 41:3 to 57:3, and 47:4 to 54:4, were observed (Figure S4 and Tables S3–S8). In the case of TAGs, the formation of abundant ammonium adduct ions [M + NH_4_]^+^ was primarily confirmed during rDUVLAESCI–MS in ESI mode. It is consistent with LC–ESI–MS analysis of human sebum.^23^ These adducts were promptly generated in positive ESI mode, as confirmed with low error in accurate mass measurement. The comparison with our rDUVLAESCI data (TAG 50:1, *m*/*z* 850.7861, −0.4 ppm; TAG 52:3, *m*/*z* 874.7824, −4.6 ppm) supports that ESI is the primary ionization process in rDUVLAESCI experiments. While the formation of these adducts was also observed during rDUVLAESCI–MS in APCI mode, protonated molecules of TAGs were significantly more prevalent (TAG 50:1, *m*/*z* 833.7605, 0.8 ppm; TAG 52:3, *m*/*z* 857.7559, 2.3 ppm), indicating the dominance of different ionization processes in the simultaneous rDUVLAESCI–MS experiment. The ionization mechanism of DAGs in rDUVLAESCI predominantly involved the dehydration of the protonated ions [M + H – H_2_O]^+^. This observation is also in correlation with previously published results of LC–ESI–MS experiments.^23^ For all of the aforementioned accurate mass measurements, cholesterol present in the sebum was used as an internal lock mass. The fragmentation spectra of the selected sebum components are presented in Figure S5. Next, exogenous compounds such as constituents of self-care cosmetics were searched for and detected in the tested latent fingerprints (Figure 5). The rDUVLAESCI spectra of the female donor contained UV filters, namely, oxybenzone (C_14_H_12_O_3_, [M + H]^+^, *m*/*z* 229.0873, 3.5 ppm), octocrylene (C_24_H_27_NO_2_, [M + H – C_8_H_18_O]^+^, *m*/*z* 232.0773, 4.7 ppm), and avobenzone (C_20_H_22_O_3_, [M + H]^+^, *m*/*z* 311.1656, 2.9 ppm), which are components of hand and face cosmetics. rDUVLAESCI–MS enables comprehensive chemical analysis from minute amounts of a sample with sufficient sensitivity for diverse groups of organic compounds. This capability positions rDUVLAESCI–MS for broad application potential, including forensic science.

### Visualization of New Psychoactive Substances on Latent Fingerprints Followed by Personal Identification

**Figure 6:**
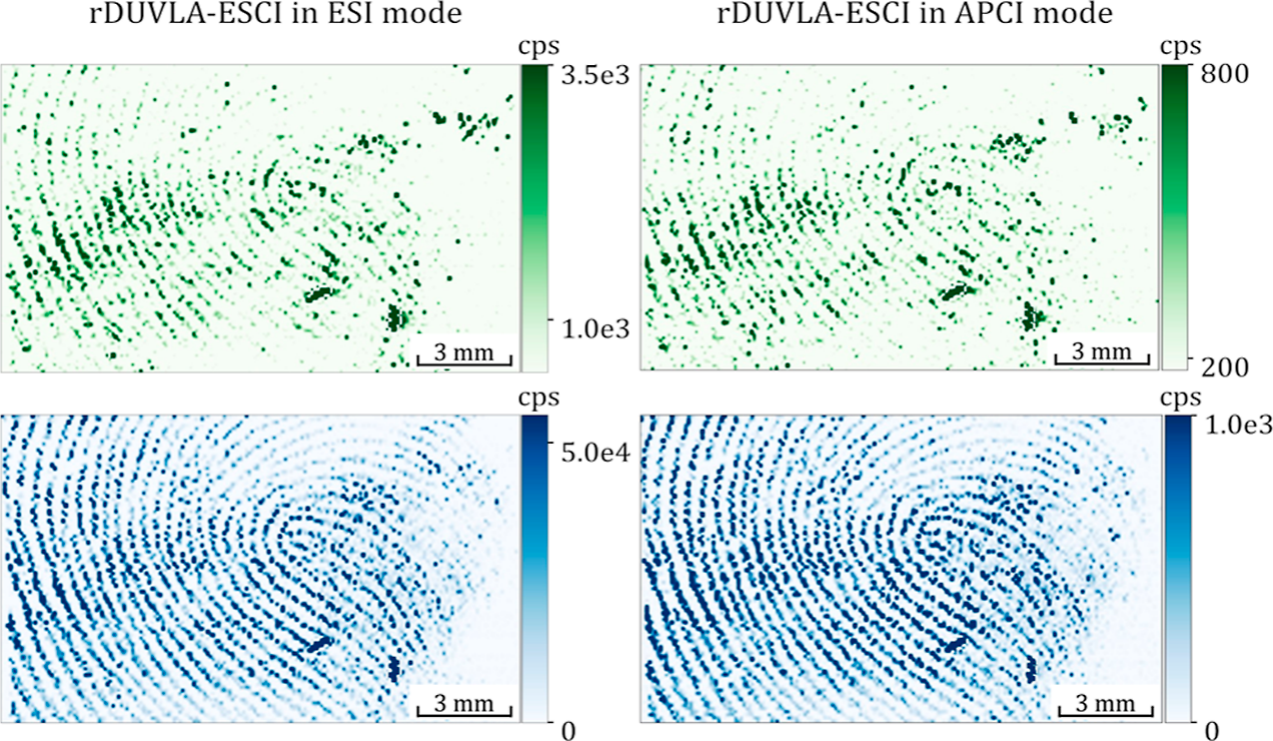
Molecular MSI of butylone and naphyrone adhered to a latent fingerprint collected from the male and female donors. rDUVLAESCI–MSI 2D images for butylone (top) and naphyrone (bottom).

**Figure 7:**
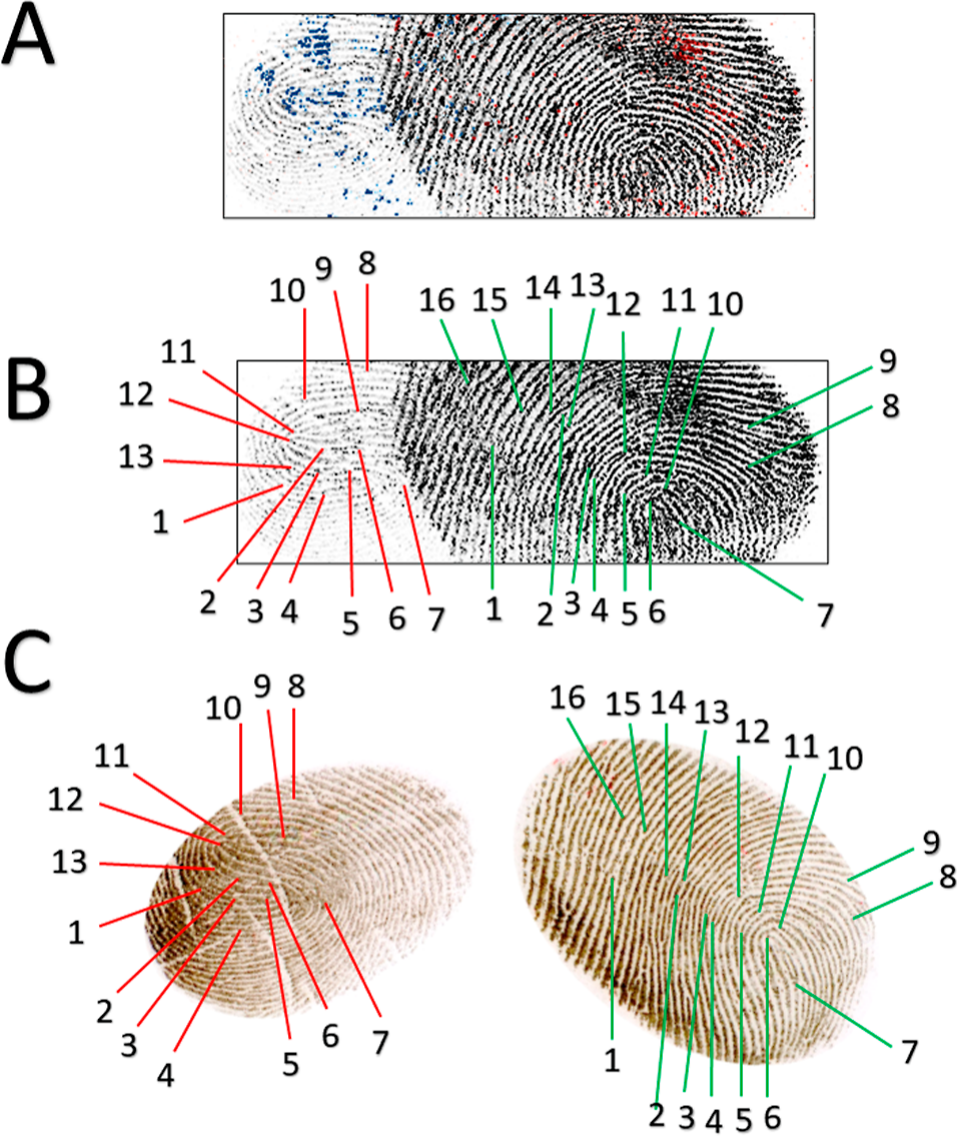
Dactyloscopic identification of the male and female donor visualizing constituents of fingerprints. 2D image: (A) co-reregistration of the cathinone and flephedrone with the fingerprint pattern; (B) palmitoyl oleate overlapped image of both persons, the thicker latent fingerprint was collected for the female donor; and (C) reference fingerprints visualized using a standard dactyloscopic approach. The positive dactyloscopic markers confirmed personal identification for both new psychoactive substances containing fingerprint donors.

The rDUVLAESCI–MSI was probed on latent fingerprints collected from donors after direct contact with either dried flephedrone and cathinone or naphyrone and butylone. The simultaneous rDUVLAESCI–MSI acquisition allowed us to visualize the spatial distribution of new psychoactive substances adhered to papillary lines (Figure 6), even in the case of the partially overlapped latent fingerprints (Figure 7). The proper optimization of the laser frequency (20 Hz), helium gas flow rate (0.65 L min^–1^), and synchronization with MS scanning frequency (acquisition of 2 spectra per pixel) enables the reconstruction of the 2D images showing reliable and non-affected papillary line patterns with negligible carryover (no sebum-related signal detected between papillary lines). Therefore, the sebum-related compounds and the new psychoactive substances (wet fingerprint) highlighted the characteristic papillary line profile that is essential for direct forensic personal identification. The reconstruction of the papillary line pattern was based on detecting the sebum constituents, namely, squalene, cholesterol, waxes, etc. (Figures 5 and 7).

The palmitoyl oleate image revealed an overlapped papillary pattern of two donors, who underwent the standard forensic fingerprint matching approach. The comparison of the characteristic dactyloscopic features provided positive matching with a visualized reference fingerprint (at least ten dactyloscopic markers are required for personal identification in Czechia and Slovakia). Even in the case of the overlapped fingerprint belonging to the donors who came into contact with flephedrone, personal identification was achieved (Figure 7). More than 15 characteristic features of overlapped fingerprints were detected. The simultaneous rDUVLAESCI–MSI renders the link between molecular features and biometric information.

## Conclusions

Our work has introduced a novel comprehensive ambient technique for complex analysis. The simultaneous acquisition of rDUVLAESCI mass spectra within a single analytical run enables the analysis of various molecules differing significantly in their polarity. The technique opens a new window in comprehensive molecular MS either for dried spot samples or for visualization of the spatial distribution of analytes. The fully automated rDUVLAESCI–MSI provided a reliable image of the molecular distribution with a variable spatial resolution in the range from 160 to 3 μm. 2D images visualizing new psychoactive substances on latent fingerprints were used for direct personal identification, even in the case of overlapping fingerprints. The successful dactyloscopic identification confirms the image quality robustness for forensic applications.

## Supporting Information Available

Full description of a rDUVLAESCI interface, rDUVLAESCI experimental conditions, rDUVLAESCI–MS results acquired at elevated laser fluence, rDUVLAESCI–MS spectra of the chemical individuals, rDUVLAESI with a variable laser focus starting from 3 μm, detailed MS, and MS/MS analysis of the fingerprint-derived sebum samples (PDF)

## References

[ref1] Venter A.; Nefliu M.; Graham Cooks R. Ambient desorption ionization mass spectrometry. TrAC, Trends Anal. Chem. 2008, 27 (4), 284–290. 10.1016/j.trac.2008.01.010.

[ref2] Feider C. L.; Krieger A.; DeHoog R. J.; Eberlin L. S. Ambient ionization mass spectrometry: recent developments and applications. Anal. Chem. 2019, 91 (7), 4266–4290. 10.1021/acs.analchem.9b00807. 30790515 PMC7444024

[ref3] Nemes P.; Vertes A. Ambient mass spectrometry for in vivo local analysis and in situ molecular tissue imaging. TrAC, Trends Anal. Chem. 2012, 34, 22–34. 10.1016/j.trac.2011.11.006.

[ref4] Vats M.; Cillero-Pastor B.; Cuypers E.; Heeren R. M. A. Mass spectrometry imaging in plants, microbes, and food: a review. Analyst 2024, 149, 4553–4582. 10.1039/d4an00644e. 39196541

[ref5] Schäfer K.-C.; Szaniszlo T.; Günther S.; Balog J.; Dénes J.; Keseru M.; Dezso B.; Tóth M.; Spengler B.; Takáts Z. In situ, real-time identification of biological tissues by ultraviolet and infrared laser desorption ionization mass spectrometry. Anal. Chem. 2011, 83 (5), 1632–1640. 10.1021/ac102613m. 21302917

[ref6] Srinivasan R. Ablation of polymers and biological tissue by ultraviolet lasers. Science 1986, 234 (4776), 559–565. 10.1126/science.3764428. 3764428

[ref7] Annangudi S. P.; Gemperline E.; Gilbert J. R. Spatial and Depth Profiling of Agricultural Formulations in Leaf Tissue Using LAESI Mass Spectrometry. J. Am. Soc. Mass Spectrom. 2024, 35 (5), 1007–1011. 10.1021/jasms.4c00026. 38613771

[ref8] Cheng C.-Y.; Yuan C.-H.; Cheng S.-C.; Huang M.-Z.; Chang H.-C.; Cheng T.-L.; Yeh C.-S.; Shiea J. Electrospray-assisted laser desorption/ionization mass spectrometry for continuously monitoring the states of ongoing chemical reactions in organic or aqueous solution under ambient conditions. Anal. Chem. 2008, 80 (20), 7699–7705. 10.1021/ac800952e. 18803395

[ref9] Kao Y. Y.; Cheng C. N.; Cheng S. C.; Ho H. O.; Shiea J. Distinguishing authentic and counterfeit banknotes by surface chemical composition determined using electrospray laser desorption ionization mass spectrometry. J. Mass Spectrom. 2013, 48 (11), 1129–1135. 10.1002/jms.3263. 24259201

[ref10] Qi K.; Lv Y.; Xiong Y.; Tian C.; Liu C.; Pan Y. Development of Transmission Ambient Pressure Laser Desorption Ionization/Postphotoionization Mass Spectrometry Imaging. Anal. Chem. 2024, 96 (14), 5489–5498. 10.1021/acs.analchem.3c05605. 38527864

[ref11] Shiea J.; Huang M.-Z.; Hsu H.-J.; Lee C.-Y.; Yuan C.-H.; Beech I.; Sunner J. Electrospray-assisted laser desorption/ionization mass spectrometry for direct ambient analysis of solids. Rapid Commun. Mass Spectrom. 2005, 19, 3701–3704. 10.1002/rcm.2243. 16299699

[ref12] Yan B.; Murta T.; Elia E. A.; Steven R. T.; Bunch J. Direct tissue mass spectrometry imaging by atmospheric pressure UV-laser desorption plasma postionization. J. Am. Soc. Mass Spectrom. 2021, 32 (2), 429–435. 10.1021/jasms.0c00315. 33289553

[ref13] Cho Y.-T.; Huang M.-Z.; Wu S.-Y.; Hou M.-F.; Li J.; Shiea J. Using electrospray laser desorption ionization mass spectrometry to rapidly examine the integrity of proteins stored in various solutions. Anal. Bioanal. Chem. 2014, 406, 577–586. 10.1007/s00216-013-7491-z. 24343451

[ref14] Lawal R. O.; Donnarumma F.; Murray K. K. Deep-ultraviolet laser ablation electrospray ionization mass spectrometry. J. Mass Spectrom. 2019, 54 (3), 281–287. 10.1002/jms.4338. 30675964 PMC6422691

[ref15] Lawal R. O.; Richardson L. T.; Dong C.; Donnarumma F.; Solouki T.; Murray K. K. Deep-ultraviolet laser ablation sampling for proteomic analysis of tissue. Anal. Chim. Acta 2021, 1184, 339021 10.1016/j.aca.2021.339021. 34625253 PMC8502231

[ref16] Sampson J. S.; Hawkridge A. M.; Muddiman D. C. Generation and detection of multiply-charged peptides and proteins by matrix-assisted laser desorption electrospray ionization (MALDESI) Fourier transform ion cyclotron resonance mass spectrometry. J. Am. Soc. Mass Spectrom. 2006, 17 (12), 1712–1716. 10.1016/j.jasms.2006.08.003. 16952462

[ref17] Xiang P.; Liyu A.; Kwon Y.; Hu D.; Williams S. M.; Veličković D. a.; Markillie L. M.; Chrisler W. B.; Paša-Tolić L.; Zhu Y. Spatial Proteomics toward Subcellular Resolution by Coupling Deep Ultraviolet Laser Ablation with Nanodroplet Sample Preparation. ACS Meas. Sci. Au 2023, 3 (6), 459–468. 10.1021/acsmeasuresciau.3c00033. 38145026 PMC10740121

[ref18] Faltusová V.; Vaculovič T.; Holá M.; Kanický V. Ilaps–python software for data reduction and imaging with LA-ICP-MS. J. Anal. At. Spectrom. 2022, 37 (4), 733–740. 10.1039/D1JA00383F.

[ref19] Stücker M.; Geil M.; Kyeck S.; Hoffman K.; Röchling A.; Memmel U.; Altmeyer P. Interpapillary lines—the variable part of the human fingerprint. J. Forensic Sci. 2001, 46 (4), 857–861. 10.1520/JFS15058J. 11451067

[ref20] van Elteren J. T.; Šelih V. S.; Šala M. Insights into the selection of 2D LA-ICP-MS (multi) elemental mapping conditions. J. Anal. At. Spectrom. 2019, 34 (9), 1919–1931. 10.1039/C9JA00166B.

[ref21] Croxton R. S.; Baron M. G.; Butler D.; Kent T.; Sears V. G. Variation in amino acid and lipid composition of latent fingerprints. Forensic Sci. Int. 2010, 199 (1–3), 93–102. 10.1016/j.forsciint.2010.03.019. 20413233

[ref22] Scruton B.; Robins B.; Blott B. The deposition of fingerprint films. J. Phys. D: Appl. Phys. 1975, 8 (6), 714 10.1088/0022-3727/8/6/016.

[ref23] Camera E.; Ludovici M.; Galante M.; Sinagra J.-L.; Picardo M. Comprehensive analysis of the major lipid classes in sebum by rapid resolution high-performance liquid chromatography and electrospray mass spectrometry [S]. J. Lipid Res. 2010, 51 (11), 3377–3388. 10.1194/jlr.D008391. 20719760 PMC2952580

