# Supplementary material for: Remote Deep-Ultraviolet Laser Ablation in Connection with Electrospray Ionization–Atmospheric Pressure Chemical Ionization (rDUVLAESCI): A Novel Dual Ionization Source for Molecular Mass Spectrometry
Source: Anal Chem. 2025 Jan 22;97(4):2062–9. doi: 10.1021/acs.analchem.4c04392 (PMC11800187; doi:10.1021/acs.analchem.4c04392)
Supplement: Supplementary file 1 — ac4c04392_si_001.pdf [file ac4c04392_si_001.pdf]

Supporting Information

Remote deep ultraviolet laser ablation in connection with electrospray ionization-atmospheric pressure chemical ionization (rDUVLAESCI): A novel dual ionization source for molecular mass spectrometry

Barbora Papoušková, Petr Fryčák, Filip Gregar, Karel Lemr, Tomáš Pluháček*

Department of Analytical Chemistry, Faculty of Science, Palacký University Olomouc, 17. listopadu 12, 77146 Olomouc, Czech Republic

*Corresponding author

Table of content	Page
Instrumentation and in-house-built rDUVLAESCI interface	S2
In-house-built rDUVLAESCI interface description	S2
Detailed study of the fragmentation degree of selected analytes using a variable laser fluence up to 12.1 $J \cdot cm^2$	S3
Optimized rDUVLAESCI-MS/MSI conditions	S8
Fragmentation spectra of sebum derived constituents	S13
Tunable rDUVLAESCI spot size (3 – 110 µm)	S14
Annotation of most abundant sebum-related compounds collected from latent fingerprints using rDUVLAESCI-MS in ESI mode and rDUVLAESCI-MS in APCI mode	S15 – S21
References	S21

Instrumentation and inhouse-built rDUVLAESCI interface

All rDUVLAESCI-MS and rDUVLAESCI-MSI experiments were carried out using an Analyte G2 remote laser ablation unit (Photon Machines, USA) fitted with a two-volume ablation chamber (100 × 100 mm y, x, z travel stage) and a nanosecond (<5 ns) 193 nm excimer laser. This setup allowed sample ablation with a variable aperture image (cross, square, round spot), spot size (for example 3 – 160 µm round spot), fluence (tenths of $J{\cdot}cm^{-2}$ –13.6 $J{\cdot}cm^{-2}$), and laser frequency (1– 300 Hz). In addition, the commercial optical system adopted the optical homogenizer of the laser beam to achieve uniform, flat surface ablation and an optical attenuator for energy control. The surplus laser energy (fluence) facilitated the complete ablation of all matrices/materials ranging from thin-layer films to glass, and metal surfaces. The Analyte G2 ablation unit was connected to a Synapt G2-S hybrid Q-TOF mass spectrometer (Waters Corporation, UK) via an in-house interface consisting of a Tygon® tube (1.2 m × 4 mm) and an x, y, z adjustable rDUVLAESCI holder designed for the commercially available ESCI Multi-mode ionization source (Chapter "Inhouse built rDUVLAESCI interface description"). The introduction of the ablated material into the center of the ESI and ESCI allowed simultaneous acquisition of both signals with a frequency of 0.3 – 0.5 s (theoretically the interscan delay starts from 15 ms).

In-house-built rDUVLAESCI interface description

The purpose of the rDUVLAESCI holder was to provide stable and adjustable positioning of the tubing that supplied the ablated material into the ion source. The holder was constructed from aluminum alloy 6063 using milling, drilling, and threading operations. The holder assembly was situated within the ion source housing and was attached to the instrument using a holder base piece that replaced the reference spray (LockSpray) holder. A rDUVLAESCI holder body was fitted onto the holder base piece and secured with a screw. Loosening this screw allowed the holder body to slide on the base, enabling vertical adjustment of the tubing tip position. The holder's arm was attached to the holder body using two screws. A tubing holder piece was seated into a groove in the holder's arm and secured with a screw. This tubing holder piece could slide in the holder arm groove, enabling horizontal adjustment of the tubing tip's position. Additionally, the tubing holder piece allowed axial adjustment of the tubing tip via two screws (**Figure S1**).

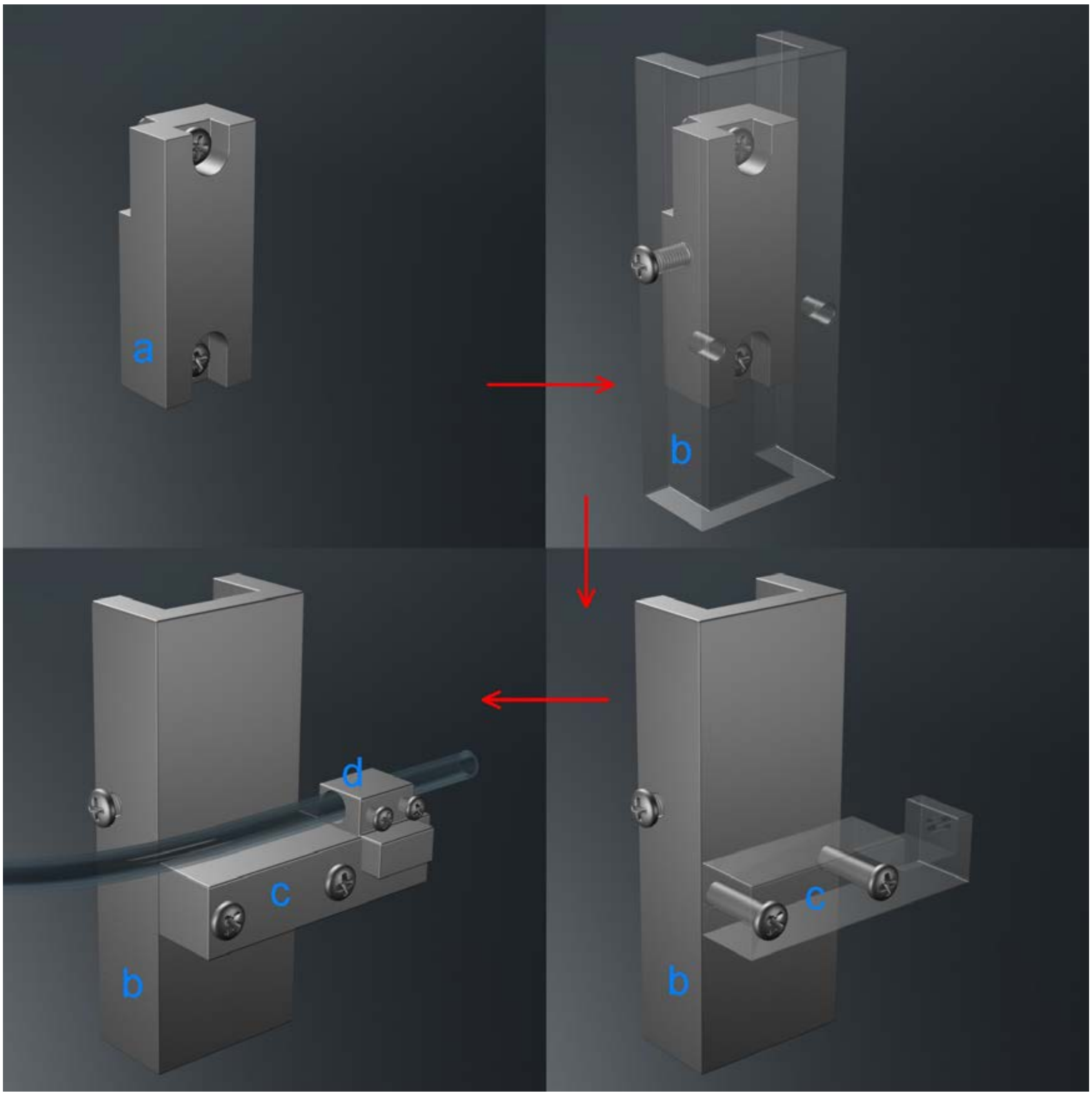

Figure S1. The scheme of the rDUVLAESCI in-house interface. rDUVLAESCI holder base (a), rDUVLAESCI holder body (b), rDUVLAESCI holder's arm (c), tubing holder (d).

Detailed study of the fragmentation degree of selected analytes using a variable laser fluence up to 12.1 J·cm^2

The determination of the fragmentation degree of selected analytes highlighted the unique behavior of rDUVLAESCI in analyzing a diverse range of organic molecules. Anthracene, caffeine, cathinone, flephedrone, mephedrone, ethyl palmitate, squalene, palmitic acid, stearic acid, theophylline, benzoic acid were analyzed by rDUVLAESCI-MS in both ESI+ and ESI- mode at the laser fluence of 1.21 - 12.1 J.cm^{-2} (Table S1). Cathinone, mephedrone, and squalene provided stable signals at the laser fluence up to 4.84 J.cm^{-2}. The most intense ion detected for the cathinones corresponded to $[M+H-H2O]^+$. This fragment ion remained detectable even at higher laser fluences, indicating that increased fluence did not affect its stability (see flephedrone). Similarly, the most intense ion for the waxes was formed by the loss of the short

alkyl chain. No significant fragmentation (greater than 13.1%) of the tested analytes was observed for the laser fluence of 1.21 $J.cm^2$, confirming that short (ns) DUV laser pulses did not extensively break down organic molecules. The similar limited fragmentation was also observed for the rDUVLAESCI-MS in APCI mode (data not shown).

Table S1. The rDUVLAESCI-MS analysis (ESI mode) with a variable laser fluence.

Fluence ($J \cdot cm^{-2}$)	**Anthracene** protonated molecule $[M+H]^+$; 179.09 *m/z* no fragment observed		**Caffeine** protonated molecule $[M+H]^+$; 195.09 *m/z* most intense fragment ($-OCNCH_3$) *m/z* 138.07 *m/z*		**Mephedrone** protonated molecule $[M+H-H_2O]^+$; 176.11 *m/z* no fragment observed	
	Fragment intensity (%)	Protonated molecule intensity, BPI* (%)	Fragment intensity (%)	Protonated molecule intensity, BPI* (%)	Fragment intensity (%)	Protonated molecule $-H_2O$ intensity, BPI* (%)
1.21	n.d.	100.0 ($6.51x10^5$)	2.0	100.0 ($1.58x10^6$)	n.d.	100.0 ($9.39x10^3$)
2.42	n.d.	100.0	1.9	100.0	n.d.	92.3
3.63	n.d.	100.0	1.7	100.0	n.d.	62.1
4.84	n.d.	100.0	1.8	100.0	n.d.	83.8
6.05	n.d.	100.0	2.7	100.0	n.d.	n.d.
7.26	n.d.	100.0	1.2	100.0	n.d.	n.d.
8.47	n.d.	100.0	1.6	100.0	n.d.	n.d.
9.68	n.d.	100.0	2.0	100.0	n.d.	n.d.
10.89	n.d.	100.0	1.9	100.0	n.d.	n.d.
12.1	n.d.	100.0	3.4	100.0	n.d.	n.d.
Fluence ($J \cdot cm^{-2}$)	**Cathinone** protonated molecule $[M+H-H_2O]^+$; 132.08 *m/z* most intense fragment ($-CH_3$) *m/z* 117.06 *m/z*		**Flephedrone** protonated molecule $[M+H-H_2O]^+$; 164.08 *m/z* most intense fragment ($-CH_3$) *m/z* 149.06 *m/z*		**Squalene** protonated molecule $[M+H-H_2O]^+$; 411.40 *m/z* no fragment observed	
	Fragment intensity (%)	Protonated molecule intensity, BPI* (%)	Fragment intensity (%)	Protonated molecule $-H_2O$, intensity, BPI* (%)	Fragment intensity (%)	Protonated molecule intensity, BPI* (%)
1.21	13.1	100.0 ($1.35x10^5$)	11.0	100.0 ($2.55x10^5$)	n.d.	66.9 ($8.94x10^4$)
2.42	15.8	100.0	12.2	100.0	n.d.	100.0
3.63	14.5	100.0	12.0	100.0	n.d.	100.0
4.84	24.3	100.0	10.5	100.0	n.d.	100.0
6.05	n.d.	n.d.	9.0	100.0	n.d.	n.d.
7.26	n.d.	n.d.	17.0	100.0	n.d.	n.d.
8.47	n.d.	n.d.	32.5	100.0	n.d.	n.d.
9.68	n.d.	n.d.	29.5	100.0	n.d.	n.d.
10.89	n.d.	n.d.	15.4	100.0	n.d.	n.d.
12.1	n.d.	n.d.	14.1	100.0	n.d.	n.d.

Table S1 continued

Fluence ($J \cdot cm^{-2}$)	**Ethyl palmitate** protonated molecule $[M+H]^+$; 285.28 *m/z* most intense fragment ($-C_2H_4$) *m/z* 257.25 *m/z*		**Palmitic acid** protonated molecule $[M+H]^+$; 257.25 *m/z* most intense fragment ($-CH_2$) *m/z* 243.23 *m/z*		**Palmitic acid** deprotonated molecule $[M-H]^-$; 283.26 *m/z* most intense fragment ($-C_2H_4$) *m/z* 255.23 *m/z*	
	Fragment intensity (%)	Protonated molecule intensity, BPI* (%)	Fragment intensity (%)	Protonated molecule intensity, BPI* (%)	Fragment intensity (%)	Deprotonated molecule intensity, BPI* (%)
1.21	100.0	73.8 ($3.14x10^5$)	3.3	4.1 ($3.08x10^4$)	2.2	100.0 ($3.92x10^5$)
2.42	100.0	72.5	3.0	3.5	1.8	100.0
3.63	100.0	74.9	2.5	3.4	1.9	100.0
4.84	100.0	69.3	2.7	5.1	2.5	100.0
6.05	100.0	65.6	10.8	23.0	5.5	100.0
7.26	100.0	65.7	19.3	27.3	8.5	100.0
8.47	100.0	63.7	23.6	46.1	14.3	100.0
9.68	100.0	61.3	18.9	39.0	15.2	100.0
10.89	100.0	63.0	33.0	53.4	15.1	100.0
12.1	100.0	62.7	33.1	67.7	15.9	49.4

Fluence ($J \cdot cm^{-2}$)	**Theophylline** protonated molecule $[M+H]^+$; 181.07 *m/z* most intense fragment (--$OCNCH_3$) *m/z* 124.05 *m/z*		**Stearic acid** protonated molecule $[M+H]^+$; 285.28 *m/z* most intense fragment ($-CH_2$) *m/z* 271.26 *m/z*		**Stearic acid** deprotonated molecule $[M-H]^-$; 283.26 *m/z* most intense fragment ($-C_2H_4$) *m/z* 255.23 *m/z*	
	Fragment intensity (%)	Protonated molecule intensity, BPI* (%)	Fragment intensity (%)	Protonated molecule intensity, BPI* (%)	Fragment intensity (%)	Deprotonated molecule intensity, BPI* (%)
1.21	2.4	100.0 ($3.01x10^5$)	7.0	11.3 ($3.59x10^5$)	5.2	100.0 ($4.70x10^5$)
2.42	4.2	100.0	8.6	11.3	5.6	100.0
3.63	3.9	100.0	8.0	11.0	5.2	100.0
4.84	3.9	100.0	9.2	9.8	4.5	100.0
6.05	4.5	100.0	6.0	9.0	3.9	100.0
7.26	5.2	100.0	7.7	9.9	5.2	100.0
8.47	3.2	100.0	12.0	11.2	5.3	100.0
9.68	2.9	100.0	10.4	11.1	8.3	100.0
10.89	1.1	100.0	26.4	20.9	10.1	100.0
12.1	14.4	100.0	53.3	34.5	6.0	100.0

Table S1 continued		
Fluence ($J \cdot cm^{-2}$)	**Benzoic acid**<br>deprotonated molecule $[M-H]^-$; 121.03 *m/z*<br>most intense fragment ($-CO_2$) *m/z* 77.04 *m/z*	
	Fragment intensity (%)	Deprotonated molecule intensity, BPI* (%)
1.21	11.7	100.0 ($6.98x10^3$)
2.42	10.4	100.0
3.63	14.2	100.0
4.84	11.0	100.0
6.05	7.3	100.0
7.26	6.6	100.0
8.47	4.7	100.0
9.68	11.5	100.0
10.89	11.1	100.0
12.1	14.0	100.0

n.d. – fragment not observed

Optimized rDUVLAESCI-MS/MSI conditions

Further experiments examined the impact of the spraying liquid flow rate on ionization mode sensitivity. It is well established that APCI and ESI differ in handling liquid flow rates and adduct formation. APCI is more suitable for higher flow rates, while ESI is typically optimized for lower flow rates to ensure efficient ionization. Additionally, ESI tends to form more adducts with ions such as sodium or ammonium, whereas APCI usually produces cleaner spectra with fewer adducts. This behavior was observed in our rDUVLAESCI source when working with flow rates, up to 200 $\mu L \cdot min^{-1}$, while analyzing a sample of olive oil spiked with ammonium ions at 1 $mmol \cdot L^{-1}$ concentration. Under these conditions, the characteristic profiles of TAGs (Fig. S4), DAGs, and FFAs were visible in APCI mode. In contrast, only ammonium adducts were detected in ESI mode. The optimal spraying liquid flow rate for both modes is typically around 5 $\mu L \cdot min^{-1}$, though this value can be adjusted in any rDUVLAESCI application to optimize SNR and enhance detection sensitivity and specificity. Consequently, both ESI and APCI modes are essential to achieve optimal analyte ionization, as demonstrated in the experimental results (**Figure S2).**

Following this, the ion source parameters and spraying liquid composition were optimized for improved performance (**Figure S3**). The optimized rDUVLAESCI conditions for ESI mode spot analyses included a laser fluence of 1.21 $J \cdot cm^{-2}$, a repetition rate of 20 Hz, and line scan ablation with a 150 µm circle spot scanned at a speed of 150 $\mu m \cdot s^{-1}$. Helium flow through the ablation chamber was set to 0.65 $L \cdot min^{-1}$. The ionization was performed in positive with a capillary voltage of 3.8 kV, source offset of 15 V, and a source temperature of 120 °C. The nebulizer gas pressure was maintained at 3.0 bar, the desolvation gas flow was set to 300 $L \cdot h^{-1}$, and the desolvation temperature was 400 °C. The spraying liquid was a mixture of MeOH:water (50:50, v/v) delivered at 5 $\mu L \cdot min^{-1}$ via a conventional ESI probe using an LC system. The mass range for analysis spanned from 50 to 1200 *m/z*, with a scan time of 0.3 s. In the negative mode, the capillary voltage was set to -2.2 kV and the source offset was increased to 80 V. These MS conditions were mirrored in APCI mode with a corona voltage of 3.5 kV for positive and -3.5 kV for negative mode to allow fast switching between ionization modes without additional adjustments.

For rDUVLAESCI mass spectrometry imaging, the method was adapted to achieve reasonable lateral resolution while maintaining the quality of the 2D maps. A 110 µm circle spot was used, scanned at a speed of 110 $\mu m \cdot s^{-1,}$ with a scan time of 0.49 s. This setup ensured synchronization between ablation pulses and mass spectrometry detection, avoiding potential quality

deterioration. The mass spectra collected during spot analyses were evaluated using MassLynx 4.1 software (Waters Corporation, UK). Time-resolved data extracted from ion traces during imaging experiments were further processed using Ilaps software, which was specifically developed for laser ablation data reduction and imaging applications. This comprehensive optimization enabled robust performance across both ionization modes and provided flexibility for a wide range of analytical applications.[1]

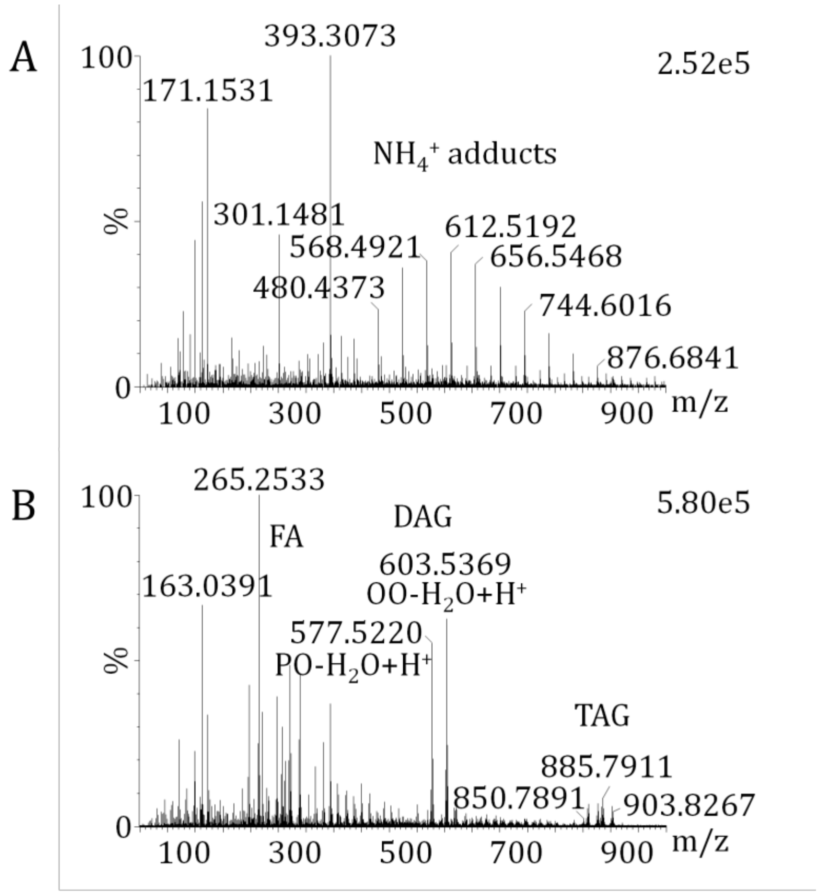

Figure S2 Evaluation of the spraying liquid flow rate (200 µL·min^{-1}) on free faty acids, diacylglycerols and triacylglycerols compound profiles in olive oil. The rDUVLAESCI-MS in ESI mode (A) and rDUVLAESCI-MS in APCI mode (B). P-palmitoyl, O-oleoyl

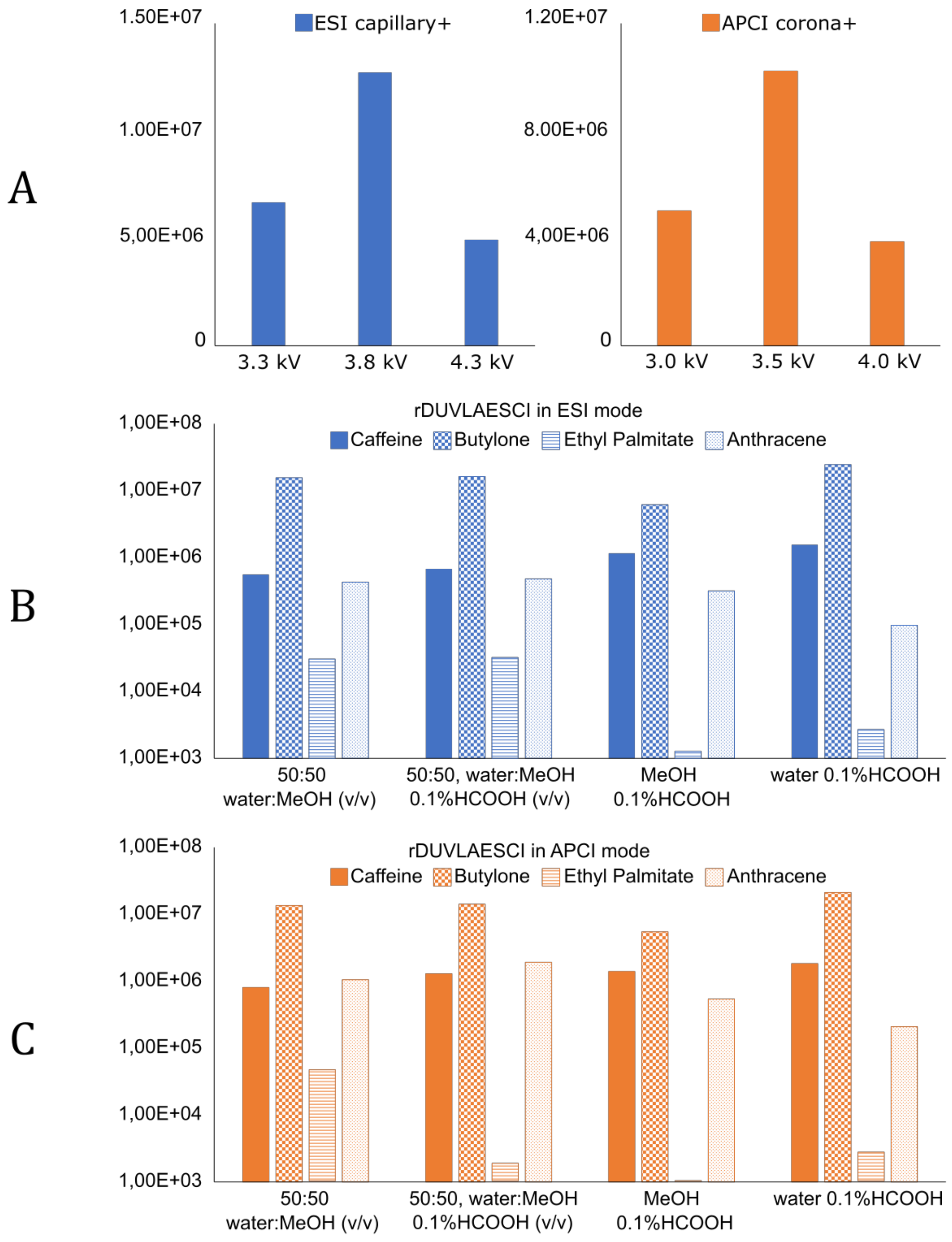

Figure S3 The optimization of the ESI and APCI (ESCI) parameters. (A) ESI capillary voltage (left) APCI corona voltage (right), (B) rDUVLAESCI in ESI mode depending on spraying liquid composition, (C) rDUVLAESCI in APCI mode depending on spraying liquid composition. The spraying liquid composition was tested using caffeine, butylone, ethyl palmitate, and anthracene.

To evaluate the major analytes from fingerprints and new psychoactive substances, the flephedrone, butylone, cathinone, squalene, and ethyl stearate were subjected to

rDUVLAESCI-MS analysis. The spectra obtained for both ionization modes are summarized in **Table S2**. The results demonstrate that simultaneous rDUVLAESI and rDUVLAAPCI signal acquisition significantly enhances organic molecule detection and improves LODs (SNR = 3) for waxes, squalene, and FFAs. Additionally, the method enabled the detection of wax esters, diacylglycerols, and triacylglycerols in thin latent fingerprint samples, highlighting its sensitivity and broad molecular coverage (**Figure S4, Table S3-Table S8**).

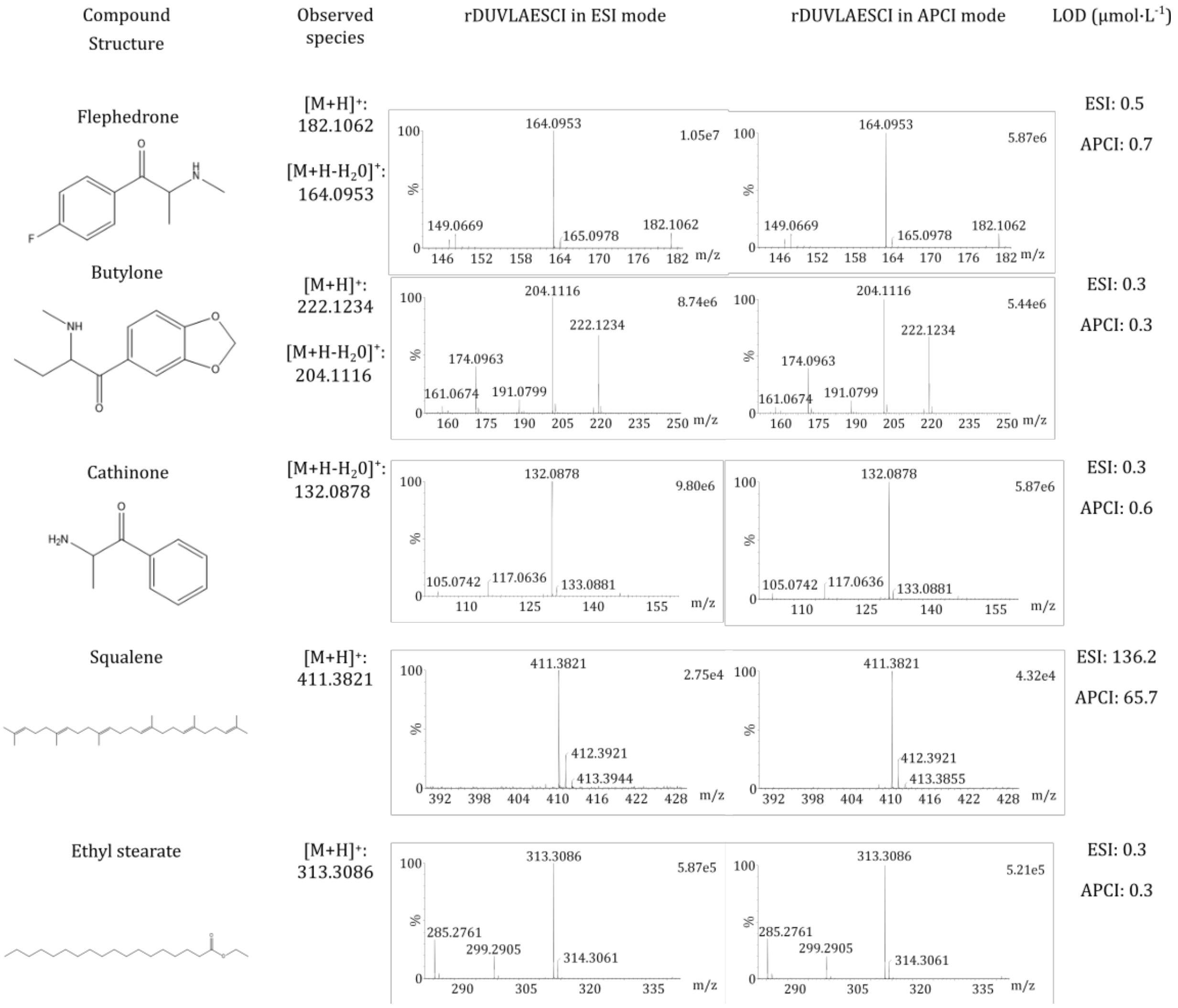

Table S2. An overview of molecules used for the novel dual source characterization together with an LOD estimation for both rDUVLAESCI-MS in ESI mode and rDUVLAESCI-MS in APCI mode.

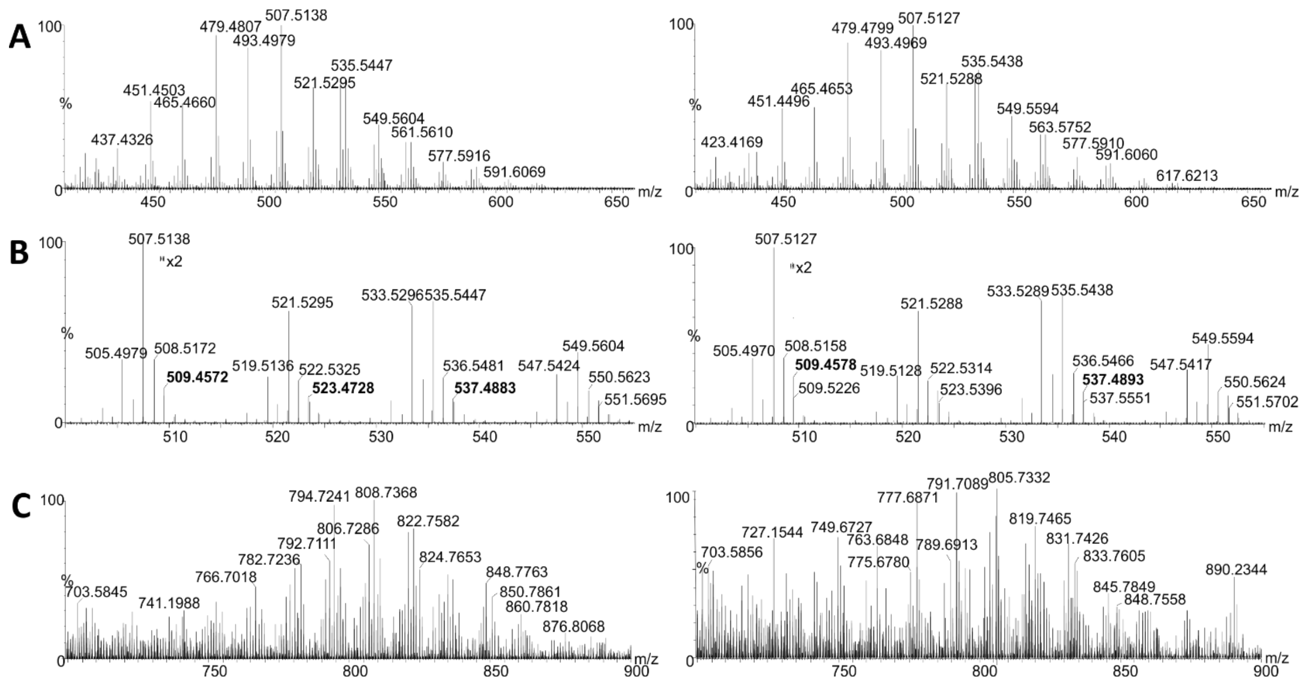

Figure S4. rDUVLAESCI-MS in ESI mode (left) and rDUVLAESCI-MS in APCI mode (right), mass spectra of wax esters (A), diacylglycerols, zoom section for DAG homologues (B), and triacylglycerols (C) in human sebum.

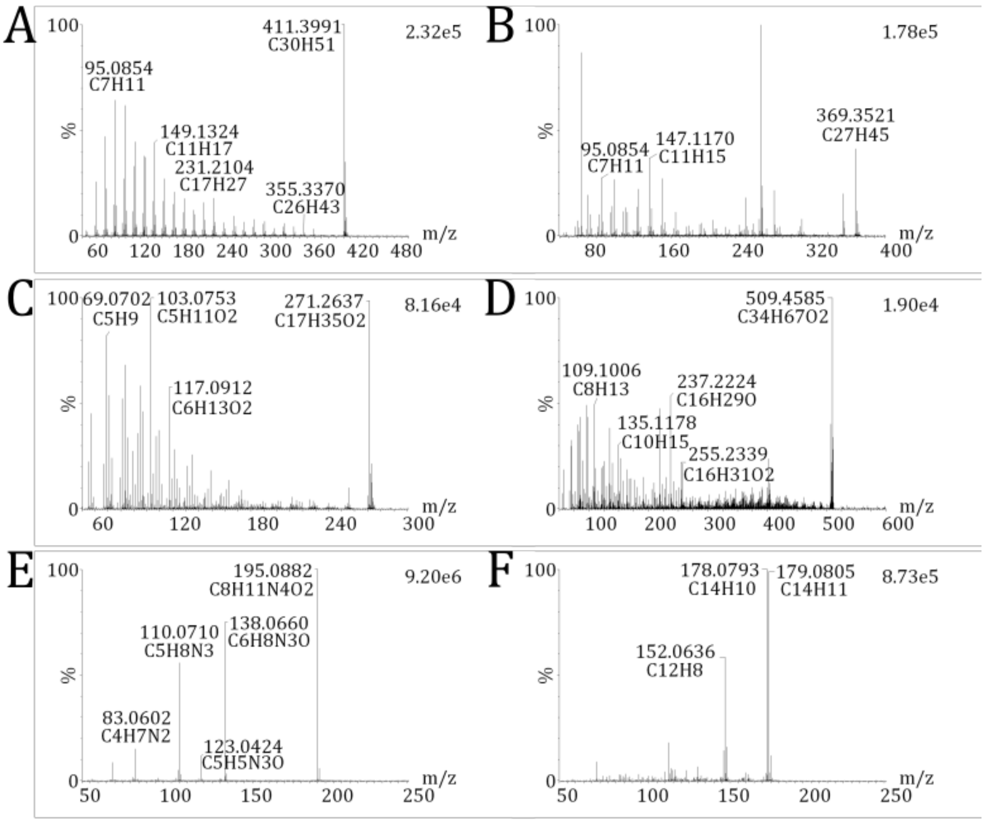

Figure S5. The fragmentation mass spectra for selected sebum constituents and model compounds acquired by rDUVLAESCI-MS in ESI mode (the APCI based MS/MS spectra followed the same fragmentation pattern, data not shown). (A) squalene, (B) cholesterol, (C) margaric acid, (D) wax ester (WE 34:1), (E) caffeine, (F) anthracene.

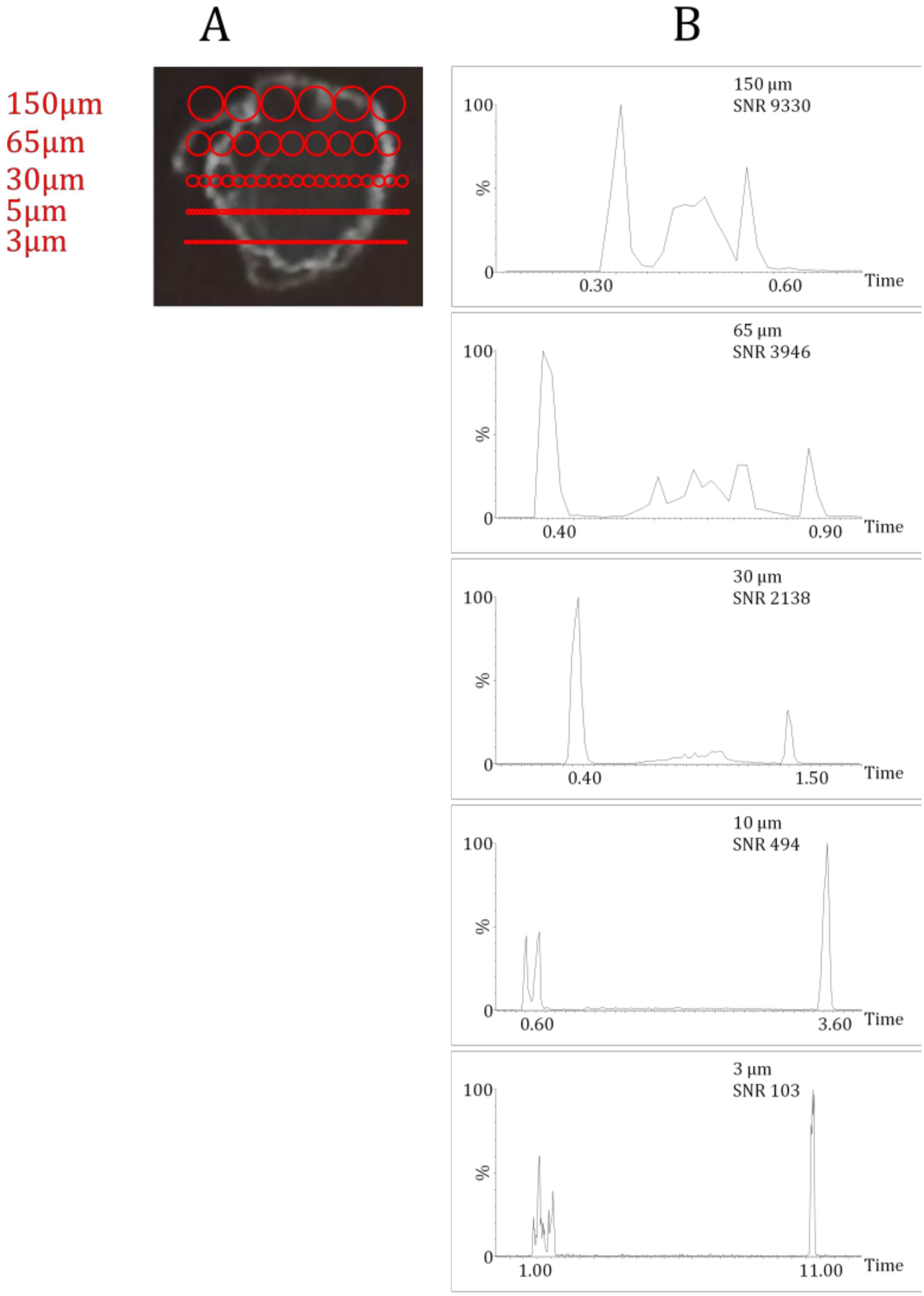

Figure S6. rDUVLAESCI-MS analysis using a tunable laser spot size. (A) dried caffeine spot (2 µL of 1 mg·ml^{-1} stock standard solution in 90:10, water: ACN (v/v), approx. 50 nmol of caffeine per 14 mm circle spot), (B) line scans probed on the dried caffeine spot with the variable laser spot size of 150, 65, 30, 10, 3 µm spot size, All measurement performed in rDUVLAESCI-MS in ESI mode (simultaneously acquired rDUVLAESCI-MS in APCI mode based line scans followed the same pattern, data not shown).

Table S3 Annotation of most abundant sebum-related wax esters collected from latent fingerprint using rDUVLAESCI-MS in ESI mode

No	Wax ester	$[M+H]^+$ formula	Calculated *m/z*	Measured *m/z*	ppm
1	WE 28:1	C28 H55 O2	423.4202	423.4172	-7.1
2	WE 29:1	C29 H57 O2	437.4359	437.4326	-7.5
3	WE 30:1	C30 H59 O2	451.4515	451.4503	-2.7
4	WE 31:1	C31 H61 O2	465.4672	465.4660	-2.6
5	WE 32:1	C32 H63 O2	479.4828	479.4807	-4.4
6	WE 33:1	C33 H65 O2	493.4985	493.4979	-1.2
7	WE 34:1	C34 H67 O2	507.5141	507.5138	-0.6
8	WE 35:1	C35 H69 O2	521.5298	521.5295	-0.6
9	WE 36:1	C36 H71 O2	535.5454	535.5447	-1.3
10	WE 37:1	C37 H73 O2	549.5611	549.5604	-1.3
11	WE 38:1	C38 H75 O2	563.5767	563.5762	-0.9
12	WE 39:1	C39 H77 O2	577.5924	577.5916	-1.4
13	WE 40:1	C40 H79 O2	591.6081	591.6069	-1.9
14	WE 41:1	C41 H81 O2	605.6231	605.6232	-0.8
15	WE 42:1	C42 H83 O2	619.6393	619.6367	-4.2
16	WE 28:2	C28 H53 O2	421.4046	421.4026	-4.7
17	WE 29:2	C29 H55 O2	435.4202	435.4181	-4.8
18	WE 30:2	C30 H57 O2	449.4359	449.4344	-3.3
19	WE 31:2	C31 H59 O2	463.4515	463.4496	-4.1
20	WE 32:2	C32 H61 O2	477.4672	477.4645	-5.7
21	WE 33:2	C33 H63 O2	491.4828	491.4814	-2.8
22	WE 34:2	C34 H65 O2	505.4985	505.4979	-1.2
23	WE 35:2	C35 H67 O2	519.5141	519.5136	-1.0
24	WE 36:2	C36 H69 O2	533.5298	533.5296	-0.4
25	WE 37: 2	C37 H71 O2	547.5454	547.5424	-5.5
26	WE 38:2	C38 H73 O2	561.5611	561.5610	-0.2
27	WE 39:2	C39 H75 O2	575.5767	575.5769	0.3
28	WE 40:2	C40 H77 O2	589.5924	589.5922	-0.3

Table S4 Annotation of most abundant sebum-related wax esters collected from latent fingerprint using rDUVLAESCI-MS in APCI mode

No	Wax ester	$[M+H]^+$ formula	Calculated *m/z*	Measured *m/z*	ppm
1	WE 28:1	C28 H55 O2	423.4202	423.4169	-7.8
2	WE 29:1	C29 H57 O2	437.4359	437.4318	-9.4
3	WE 30:1	C30 H59 O2	451.4515	451.4496	-4.2
4	WE 31:1	C31 H61 O2	465.4672	465.4653	-4.1
5	WE 32:1	C32 H63 O2	479.4828	479.4799	-6.0
6	WE 33:1	C33 H65 O2	493.4985	493.4969	-3.2
7	WE 34:1	C34 H67 O2	507.5141	507.5127	-2.8
8	WE 35:1	C35 H69 O2	521.5298	521.5288	-1.9
9	WE 36:1	C36 H71 O2	535.5454	535.5438	-3.0
10	WE 37:1	C37 H73 O2	549.5611	549.5594	-3.1
11	WE 38:1	C38 H75 O2	563.5767	563.5752	-2.7
12	WE 39:1	C39 H77 O2	577.5924	577.5910	-2.4
13	WE 40:1	C40 H79 O2	591.6081	591.6060	-3.4
14	WE 41:1	C41 H81 O2	605.6231	605.6214	-3.8
15	WE 42:1	C42 H83 O2	619.6393	619.6359	-5.5
16	WE 28:2	C28 H53 O2	421.4046	423.4018	-6.6
17	WE 29:2	C29 H55 O2	435.4202	437.4170	-7.3
18	WE 30:2	C30 H57 O2	449.4359	449.4333	-5.8
19	WE 31:2	C31 H59 O2	463.4515	463.4487	-6.0
20	WE 32:2	C32 H61 O2	477.4672	477.4637	-7.3
21	WE 33:2	C33 H63 O2	491.4828	491.4807	-4.3
22	WE 34:2	C34 H65 O2	505.4985	505.4970	-3.0
23	WE 35:2	C35 H67 O2	519.5141	519.5128	-2.5
24	WE 36:2	C36 H69 O2	533.5298	533.5289	-1.7
25	WE 37: 2	C37 H71 O2	547.5454	547.5417	-6.8
26	WE 38:2	C38 H73 O2	561.5611	561.5600	-2.0
27	WE 39:2	C39 H75 O2	575.5767	575.5754	-2.3
28	WE 40:2	C40 H77 O2	589.5924	589.5915	-1.5
29	WE 41:2	C41 H79 O2	603.6080	603.6064	-2.7
30	WE 42:2	C42 H81 O2	617.6237	617.6213	-3.9

Table S5 Annotation of most abundant sebum-related diacylglycerols collected from latent fingerprint using rDUVLAESCI-MS in ESI mode

No	DAG	$[M+H-H_2O]^+$ formula	Calculated *m/z*	Measured *m/z*	ppm
1	DAG 29:0	C32 H61 O4	509.4570	509.4572	0.4
2	DAG 30:0	C33 H63 O4	523.4726	523.4728	0.4
3	DAG 31:0	C34 H65 O4	537.4883	537.4883	0.0
4	DAG 32:0	C35 H67 O4	551.5039	551.5042	0.5
5	DAG 29:1	C32 H59 O4	507.4413	507.4402	-2.2
6	DAG 30:1	C33 H61 O4	521.4570	521.4567	-0.6
7	DAG 31:1	C34 H63 O4	535.4726	535.4717	-1.7
8	DAG 32:1	C35 H65 O4	549.4883	549.4882	-0.2
9	DAG 29:2	C32 H57 O4	505.4257	505.4243	-2.8
10	DAG 30:2	C33 H59 O4	519.4413	519.4400	-2.5
11	DAG 31:2	C34 H61 O4	533.4570	533.4552	-3.4
12	DAG 32:2	C35 H63 O4	547.4726	547.4695	-5.7
13	DAG 29:3	C32 H55 O4	503.4100	503.4085	-3.0

Table S6 Annotation of most abundant sebum-related diacylglycerols collected from latent fingerprint using rDUVLAESCI-MS in APCI mode

No	DAG	$[M+H-H_2O]^+$ formula	Calculated *m/z*	Measured *m/z*	ppm
1	DAG 29:0	C32 H61 O4	509.4570	509.4578	1.6
2	DAG 30:0	C33 H63 O4	523.4726	523.4734	1.5
3	DAG 31:0	C34 H65 O4	537.4883	537.4893	1.9
4	DAG 32:0	C35 H67 O4	551.5039	551.5050	2.0
5	DAG 29:1	C32 H59 O4	507.4413	507.4408	-1.0
6	DAG 30:1	C33 H61 O4	521.4570	521.4556	-2.7
7	DAG 31:1	C34 H63 O4	535.4726	535.4718	-1.5
8	DAG 32:1	C35 H65 O4	549.4883	549.4881	-0.4
9	DAG 29:2	C32 H57 O4	505.4257	505.4222	-6.9
10	DAG 30:2	C33 H59 O4	519.4413	519.4419	1.2
11	DAG 31:2	C34 H61 O4	533.4570	533.4539	-5.8
12	DAG 32:2	C35 H63 O4	547.4726	547.4710	-2.9
13	DAG 29:3	C32 H55 O4	503.4100	503.4085	-3.0

Table S7 Annotation of most abundant sebum-related triacylglycerols collected from latent fingerprint using rDUVLAESCI-MS in ESI mode

**No**	**TAG**	**$[M+NH_4]^+$ formula**	**Calculated *m/z***	**Measured m/z**	**ppm**
1	TAG 40:0	C43 H86 N O6	712.6455	712.6435	-2.8
2	TAG 41:0	C44 H88 N O6	726.6612	----	
3	TAG 42:0	C45 H90 N O6	740.6768	740.6771	0.4
4	TAG 43:0	C46 H92 N O6	754.6925	754.6890	-4.6
5	TAG 44:0	C47 H94 N O6	768.7081	768.7133	6.8
6	TAG 45:0	C48 H96 N O6	782.7238	782.7236	-0.3
7	TAG 46:0	C49 H98 N O6	796.7394	796.7466	9.0
8	TAG 47:0	C50 H100 N O6	810.7551	810.7631	9.9
9	TAG 48:0	C51 H102 N O6	824.7707	824.7653	-6.5
10	TAG 49:0	C52 H104 N O6	838.7864	838.7900	4.3
11	TAG 50:0	C53 H106 N O6	852.8020	852.8015	-0.6
12	TAG 51:0	C54 H108 N O6	866.8177	866.8214	4.2
13	TAG 52:0	C55 H110 N O6	880.8333	----	
14	TAG 53:0	C56 H112 N O6	894.8490	----	
15	TAG 40:1	C43 H84 N O6	710.6299	710.6338	5.5
16	TAG 41:1	C44 H86 N O6	724.6455	724.6517	8.6
17	TAG 42:1	C45 H88 N O6	738.6612	738.6658	6.2
18	TAG 43:1	C46 H90 N O6	752.6768	752.6825	7.6
19	TAG 44:1	C47 H92 N O6	766.6925	766.7018	12.1
20	TAG 45:1	C48 H94 N O6	780.7081	780.7090	1.2
21	TAG 46:1	C49 H96 N O6	794.7238	794.7241	0.4
22	TAG 47:1	C50 H98 N O6	808.7394	808.7368	-3.2
23	TAG 48:1	C51 H100 N O6	822.7551	822.7582	3.8
24	TAG 49:1	C52 H102 N O6	836.7707	836.7637	-8.4
25	TAG 50:1	C53 H104 N O6	850.7864	850.7888	2.8
26	TAG 51:1	C54 H106 N O6	864.8020	864.8051	3.6
27	TAG 52:1	C55 H108 N O6	878.8177	----	
28	TAG 53:1	C56 H110 N O6	892.8333	892.8262	-8.0
29	TAG 40:2	C43 H82 N O6	708.6142	708.6121	-3.0
30	TAG 41:2	C44 H84 N O6	722.6299	722.6242	-7.9
31	TAG 42:2	C45 H86 N O6	736.6455	736.6457	0.3
32	TAG 43:2	C46 H88 N O6	750.6612	----	
33	TAG 44:2	C47 H90 N O6	764.6768	----	
34	TAG 45:2	C48 H92 N O6	778.6925	778.6907	-2.3
35	TAG 46:2	C49 H94 N O6	792.7081	792.7111	3.8
36	TAG 47:2	C50 H96 N O6	806.7238	806.7286	5.9
37	TAG 48:2	C51 H98 N O6	820.7394	820.7463	8.4
38	TAG 49:2	C52 H100 N O6	834.7551	834.7493	-6.9
39	TAG 50:2	C53 H102 N O6	848.7707	848.7763	6.6
40	TAG 51:2	C54 H104 N O6	862.7864	862.7889	2.9

Table S7 (continued)					
**No**	**TAG**	**$[M+NH_4]^+$ formula**	**Calculated *m/z***	**Measured *m/z***	**ppm**
41	TAG 52:2	C55 H106 N O6	876.8020	876.8068	5.5
42	TAG 53:2	C56 H108 N O6	890.8177	890.8130	-5.3
43	----				
44	TAG 41:3	C44 H82 N O6	720.6142	720.6142	6.8
45	TAG 42:3	C45 H84 N O6	734.6299	734.6230	-9.4
46	TAG 43:3	C46 H86 N O6	748.6455	748.6512	7.6
47	TAG 44:3	C47 H88 N O6	762.6612	762.6619	0.9
48	TAG 45:3	C48 H90 N O6	776.6768	776.6698	-9.0
49	TAG 46:3	C49 H92 N O6	790.6925	790.6943	2.3
50	TAG 47:3	C50 H94 N O6	804.7081	804.7088	0.9
51	TAG 48:3	C51 H96 N O6	818.7238	818.7238	-1.7
52	TAG 49:3	C52 H98 N O6	832.7394	832.7455	7.3
53	TAG 50:3	C53 H100 N O6	846.7551	846.7618	7.9
54	TAG 51:3	C54 H102 N O6	860.7707	860.7704	-0.3
55	TAG 52:3	C55 H104 N O6	874.7864	874.7878	1.6
56	TAG 53:3	C56 H106 N O6	888.8020	----	

Table S8 Annotation of most abundant sebum-related triacylglycerols collected from latent fingerprint using rDUVLAESCI-MS in APCI mode

No	TAG	$[M+H]^+$ formula	Calculated *m/z*	Measured *m/z*	ppm
1	TAG 43:0	C46 H89 O6	737.6737	----	
2	TAG 44:0	C47 H91 O6	751.6816	----	
3	TAG 45:0	C48 H93 O6	765.6972	----	
4	TAG 46:0	C49 H95 O6	779.7129	----	
5	TAG 47:0	C50 H97 O6	793.7285	----	
6	TAG 48:0	C51 H99 O6	807.7520	807.7410	-4.0
7	TAG 49:0	C52 H101 O6	821.7598	----	
8	TAG 50:0	C53 H103 O6	835.7755	----	
9	TAG 51:0	C54 H105 O6	849.7911	----	
10	TAG 52:0	C55 H107 O6	863.8068	----	
11	TAG 53:0	C56 H109 O6	877.8224	----	
12	TAG 43:1	C46 H87 O6	735.6503	735.6539	4.9
13	TAG 44:1	C47 H89 O6	749.6659	749.6727	9.1
14	TAG 45:1	C48 H91 O6	763.6816	763.6848	4.2
15	TAG 46:1	C49 H93 O6	777.6972	----	
16	TAG 47:1	C50 H95 O6	791.7129	791.7089	-5.1
17	TAG 48:1	C51 H97 O6	805.7285	----	
18	TAG 49:1	C52 H99 O6	819.7442	819.7465	2.8
19	TAG 50:1	C53 H101 O6	833.7598	833.7605	0.8
20	TAG 51:1	C54 H103 O6	847.7755	847.7687	-8.0
21	TAG 52:1	C55 H105O6	861.7911	----	
22	TAG 53:1	C56 H107 O6	875.8086	----	
23	TAG 43:2	C46 H85 O6	733.6346	----	
24	TAG 44:2	C47 H87 O6	747.6503	----	
25	TAG 45:2	C48 H89 O6	761.6659	761.6732	9.6
26	TAG 46:2	C49 H91 O6	775.6816	775.6780	-4.6
27	TAG 47:2	C50 H93 O6	789.6972	789.6913	-7.5
28	TAG 48:2	C51 H95 O6	803.7129	----	
29	TAG 49:2	C52 H97 O6	817.7285	817.7340	6.7
30	TAG 50:2	C53 H99 O6	831.7442	831.7426	-1.9
31	TAG 51:2	C54 H101 O6	845.7598	845.7618	2.4
32	TAG 52:2	C55 H103 O6	859.7755	859.7817	7.2
33	TAG 53:2	C56 H105 O6	873.7911	873.7935	2.7
34	TAG 54:2	C57 H107 O6	887.8068	887.8006	-7.0
35	TAG 51:2	C54 H101 O6	845.7598	845.7618	2.4
36	TAG 52:2	C55 H103 O6	859.7755	859.7817	7.2
37	TAG 53:2	C56 H105 O6	873.7911	873.7935	2.7
38	TAG 54:2	C57 H107 O6	887.8068	887.8006	-7.0
39	TAG 43:3	C46 H83 O6	731.6190	----	
40	TAG 44:3	C47 H85 O6	745.6346	----	

Table S8 (continued)					
**No**	**TAG**	**$[M+H]^+$ formula**	**Calculated *m/z***	**Measured m/z**	**ppm**
41	TAG 45:3	C48 H87 O6	759.6503	759.6461	-5.5
42	TAG 46:3	C49 H89 O6	773.6659	----	
43	TAG 47:3	C50 H91 O6	787.6818	----	
44	TAG 48:3	C51 H93 O6	801.6972	801.6940	-4.0
45	TAG 49:3	C52 H95 O6	815.7129	815.7200	8.7
46	TAG 50:3	C53 H97 O6	829.7285	----	
47	TAG 51:3	C54 H99 O6	843.7442	843.7502	7.1
48	TAG 52:3	C55 H101 O6	857.7598	857.7642	5.1
49	TAG 53:3	C56 H103 O6	871.7755	871.7736	-2.2
50	TAG 54:3	C57H105 O6	885.7911	885.7883	-3.2

References

(1) Faltusová, V.; Vaculovič, T.; Holá, M.; Kanický, V. Ilaps–python software for data reduction and imaging with LA-ICP-MS. *Journal of Analytical Atomic Spectrometry* **2022**, *37* (4), 733-740.
